# Regulation of salt tolerance in the roots of *Zea mays* by L-histidine through transcriptome analysis

**DOI:** 10.3389/fpls.2022.1049954

**Published:** 2022-11-28

**Authors:** Hongfei Ji, Guoping Yang, Xiu Zhang, Qiumei Zhong, Yuxi Qi, Kaihua Wu, Tingting Shen

**Affiliations:** Ningxia Key Laboratory for the Development and Application of Microbial Resources in Extreme Environments, College of Biological Science and Engineering, North Minzu University, Yinchuan, China

**Keywords:** *Zea mays*, enzymatic activity, transcriptomic, L-histidine, salt stress

## Abstract

Soil salinization is an important worldwide environmental problem and the main reason to reduce agricultural productivity. Recent findings suggested that histidine is a crucial residue that influences the ROS reduction and improves the plants’ tolerance to salt stress. Herein, we conducted experiments to understand the underlying regulatory effects of histidine on maize root system under salt stress (100 mM NaCl solution system). Several antioxidant enzymes were determined. The related expressed genes (DEGs) with its pathways were observed by Transcriptome technologies. The results of the present study confirmed that histidine can ameliorate the adverse effects of salt stress on maize root growth. When the maize roots exposed to 100 mM NaCl were treated with histidine, the accumulation of superoxide anion radicals, hydrogen peroxide, and malondialdehyde, and the content of nitrate nitrogen and ammonium nitrogen were significantly reduced; while the activities of superoxide dismutase, peroxidase, catalase, nitrate reductase, glutamine synthetase, and glutamate synthase were significantly increased. Transcriptome analysis revealed that a total of 454 (65 up-regulated and 389 down-regulated) and 348 (293 up-regulated and 55 down-regulated) DEGs were observed when the roots under salt stress were treated with histidine for 12 h and 24 h, respectively. The pathways analysis of those DEGs showed that a small number of down-regulated genes were enriched in phytohormone signaling and phenylpropanoid biosynthesis at 12 h after histidine treatment, and the DEGs involved in the phytohormone signaling, glycolysis, and nitrogen metabolism were significantly enriched at 24 h after treatment. These results of gene expression and enzyme activities suggested that histidine can improve the salt tolerance of maize roots by enriching some DEGs involved in plant hormone signal transduction, glycolysis, and nitrogen metabolism pathways.

## Introduction

Soil salinization is one of the most well-known threats to the development of global agriculture. The Food and Agriculture Organization of the United Nations estimates that salt has affected more than 6% of the land area (Ilangumaran and Smith, 2017), and about 20% of the world’s irrigated land is impacted by salt, with a direct economic loss of $12 billion per year (Tang et al., 2014). The monitoring of soil salinity and land cover revealed that Asia is one of the continents most affected by salt, especially the northwest of China (Hassani et al., 2020). The total area of saline soil in China is about 3.6×10^7^ ha, accounting for 4.88% of the country’s total available land base. The excessive accumulation of water-soluble salts in soil, such as K^+^, Mg^2+^, Ca^2+ ^, Cl^-^, SO_4_^2-^, CO_3_^2-^, HCO_3_^-^, Na^+^ plasma, etc., can damage the soil structure, produce toxic effects on plants, hinder the growth of crops, cause the decline of soil fertility, and seriously reduce the productivity of the land (Parida and Das, 2005).

Salinization of soil results from a combination of evaporation, salt precipitation and dissolution, salt transport, and ion exchange. Excessive soluble salt in the soil can lead to plant salt stress, one of the most detrimental environmental stresses, osmotic stress, ionic toxicity, and oxidative stress (Bojórquez-Quintal et al., 2014; Tanveer and Shabala, 2018). High salt stress can increase the levels of the reactive oxygen species (ROS) and result in oxidative stress, which in turn affects the plants both at cellular and metabolic levels (Duan et al., 2015).

ROS play important roles in maintaining normal plant growth and improving their tolerance to environmental stresses. It has been implicated as second messengers in plant hormone responses and function as important signaling molecules that regulate normal plant growth and response hormones to stresses, such as salt stress (Yu et al., 2020). At high levels, active oxygen species can lead to impaired physiological function through cellular damage and oxidization of DNA, protein, and lipid membrane of plant cells. For example, the accumulation of Ca^2+^ can activate the ROS signals and change their phospholipid components, induce plant hormone signal transmission, and regulate cytoskeleton dynamics and cell wall structure, which will slow down the plant root growth and increase the metabolites (Bybordi, 2010; Zhao et al., 2021).

Nitrogen is an essential element in plant growth. Plant absorbs nitrate from soil and transforms it into amino acids through a series of assimilation processes such as nitrogen metabolism. These processes are affected by abiotic stress, especially salt stress. The change of enzyme activity in nitrogen metabolism depends on the species of plants and the sensitivity of plants to salt stress (Gouia et al., 1994; Li et al., 2019; Yin et al., 2020). In addition, salt stress also affects sugar metabolism, changes sugar levels, such as sucrose and fructose, and causes changes in enzyme activity in glycolysis (Shumilina et al., 2009). The hormones in plant play an important role in mediating salt stress signals and controlling the balance between growth and salt stress response. Plants will regulate growth and development through hormone signal transduction and improve the adaptability to salt stress (Yu et al., 2020).

Root is the first organ to undergo salinity stress and plays an important role in salt sensing. Root system can dynamically adjust its development and performance in response to biotic and abiotic stresses, including modulation of root growing, branching, forking, and redirecting. These responses can be manifested differentially at the cellular, tissue, or organ levels. To fully capture the responses of root system to salt stress, it is critical to explore the key genes and associated functions for development of salt tolerance in crops.

L-histidine is one of the standard amino acids in proteins and critical for plant growth and development. Histidine kinases (HK) play important roles in the regulation of plant development in response to hormones, as well as environmental stimuli (Nongpiur et al., 2012). The study of the interaction between histidine and membranes and macromolecules confirmed that histidine plays a unique role in enabling protein/peptide-membrane interactions that occur in marine or other high-salt environments (Xian et al., 2022). Previous studies also showed that histidine takes an important role in regulating the biosynthesis of other amino acids, the chelation transport of metal ions, and the development and growth of plant embryos in different plants, including rice (*Arabidopsis)* and maize (*Zea mays)* (Radwanski and Last, 1995; Kramer et al., 1996; Noutoshi et al., 2005; Stepansky and Leustek, 2006; Irtelli et al., 2009). However, the genetic effects of salinity on maize’s root development at the gene level remain unclear and the genetic machinery underlying maize root responses to salt stress remains uncharacterized.

Maize is the most widely planted grain in the world and ranked second in the important crops of China, which accounts for 29.5% of total grain production (Medeiros et al., 2021). Soil salinization is one of the major abiotic stresses negatively impacting growth, development, yield, and seed quality of maize production. The critical challenge for enhancing the quality and productivity of maize is how to improve its tolerance or to incorporate resistance to different stresses, including salt stress.

In the present study, maize seeds were grown in lab. After gemination, we treated the seedling roots with salt stress and histidine for different time periods to 1) compare the natural traits and morphological variations of seedling roots among the control and treatment samples, 2) evaluate the antioxidant roles and activities of histidine in antioxidant enzymes and nitrogen metabolism in the seedling roots under salt stress, 3) identify differentially expressed genes under control, salt stress, and histidine treatment conditions by RNA-seq analysis, and 4) investigate the effects of histidine on the potential pathways responsible for differences in root salt stress responses by gene co-expression network and pathway analyses. Our study will provide accurate quantitative analysis of an effective treatment of soil salinization and scientific guidance for the future formulation of salinization control in crops.

## Materials and methods

### Plant materials and treatments

All plant materials used in the experiments were from the maize variety “ningdan 33”. All experiments were conducted at the Ningxia Key Laboratory for the Development and Application of Microbial Resources in Extreme Environments, North Minzu University, China. The seeds from the variety were disinfected with 0.1% HgCl_2_ and inoculated in 0.8% agar medium (containing hoagland nutrient solution) supplemented with sterile water and 0.1 μM histidine for 12 h at 4°C. The samples were retained in a box with the relative humidity at 75% and the temperature at 28°C. When seed roots grew up to 3 cm, all samples were randomly divided into 4 groups, including (i) the non-stress control (CK0) treated with hoagland nutrient solution, (ii) the non-stress treatment group (T0) treated with 0.1 μM histidine+hoagland nutrient solution, (iii) the salt stress control (CK1) treated with100 mM NaCl+hoagland nutrient solution, and (iv) the salt stress treatment group (T1) treated with 0.1 μM histidine+100 mM NaCl+hoagland nutrient solution. Each group has 15 hydroponic plastic bottles of 4 seeds and 450g sterilized small stones each. The incubator was set with the relative humidity 75% and conditions of 13,000 LX at 28°C for 4 h, 11,000 LX at 25°C for 4 h, 9,000 LX at 20°C for 3 h, 6,000 LX at 20°C for 2 h, 9,000 LX, at 25°C for 3 h and dark at 18°C for 8 h.

Our previous experiment confirmed the alleviating effect of the histidine at 100 μM, 10 μM, 1 μM, 0.1 μM, 0.01 μM, 0.001 μM on the NaCl induced damage to the root, with the treatment of 0.1 μM of histidine resulted in the most substantial resistance in the maize seedling roots (**Figure S1**). After treatment for 12 h and 24 h, 12 young roots were randomly selected and divided into three sub-groups at each time point. All samples were snap-frozen with liquid nitrogen and stored at -80°C. RNA was extracted from each sample for transcriptomic analysis. After 7 days treatment, the root tips were stained with DAB and NBT. After 14 days treatment, the total root length, root projected area, root volume, root surface area, numbers of root tips, and forks from each treatment were measured with an Analyzer (GXY-A, Sichuan, China), and the activities of root related enzymes were simultaneously measured.

### Nitroblue tetrazolium and diaminobenzidine staining of root tips

The DBA reaction was conducted in the dark at 28 °C for 6 h following the method of Li et al. (2013). The root tips of each treatment group were collected and plated into the culture plate with 1mg ml^-1^ DAB reaction solution (pH 5.5, 50 mM Tris HCl). The samples were then transferred to 90% (v/v) ethanol for decolorization in a 70 °C water bath and stored in 50% glycerol. The highly localized accumulation of H_2_O_2_ was observed as dark brown at X100 magnification. The NBT staining was based on the method of Khokon et al. (2011). The root tips of each treatment were placed into the culture plate with adding 0.5 mg ml^-1^ NBT reaction solution (pH 7.8, 50 mm PBS). The culture plates were kept in dark at 28°C for 4 h, and then transferred to a 70°C water bath containing 90% (v/v) ethanol for decolorization and stored in 50% glycerol. The localization of O_2_^‐^ was observed as blue at X100 magnification.

### Measurement of O_2_·^‐^and H_2_O_2_ content

The contents of O_2_^‐^ and H_2_O_2_ of the samples were determined following the method of Jambunathan (2010). One gram of the frozen root tissue of each sample mixed with 5mL of 50 mM PBS (pH 7.8) was grinded and homogenized by centrifugation. One mL of extraction solution of each sample was mixed with 0.5 mL PBS and 10 mM hydroxylamine hydrochloride, and then reacted at 25°C for 30 min. After added 1 mL of 17 mM p-Aminobenzene Sulfonic Acid and α-Naphthylamine, the mixture was incubated at 25°C for 15 min. The absorbance value was measured at 530 nm and the content of O_2_^‐^ was calculated based on the standard curve.

The extraction solution for determining the contents of H_2_O_2_ was processed by using acetone grinding and homogenization by centrifuging. One mL of the extraction solution was mixed with 0.1 mL of 5% titanium sulfate (v/v) and 0.2 mL of strong aqua ammonia and precipitated. After discarded the supernatant, 5 mL of 2 M sulphuric acid was added in the mixture, and the absorbance value was read at 415 nm and the H_2_O_2_ content was determined with the standard curve.

### Measurement of the contents of ammonium nitrogen and nitrate nitrogen

The method of Cataldo et al. (1975) was referred for determining the content of nitrate nitrogen (NO_3_^-^) in plants. One gram of the frozen root tissue of each sample was extracted in a distilled boiling water bath for 30 min. After cooled, the extraction solution was diluted with distilled water to 25 mL. Then 0.1 ml of the diluted extraction solution was mixed with 0.4 mL salicylic acid (H_2_SO_4_^-^ [1:5 (v/v)]) for 20 min. After addition of 9.5 mL of 2 M NaOH, the absorbance value of the solution was read at 410 nm and the NO_3_^-^ content was calculated with the standard curve.

The content of ammonium nitrogen (NH_4_^+^) was determined by a ninhydrin colorimetry. Half of a gram of the frozen root tissue of each sample was mixed with 1.7 M acetic acid and diluted with distilled water to 100 mL. Two mL of the diluted extraction solution was mixed with 3 mL of 54 mM acidic ninhydrin acetic acid buffer (pH 5.4) and 0.1 mL of 60 mM ascorbic acid, and incubated in a boiling water bath for 15 min. After cooled, absolute ethanol was added to 10 mL. The absorbance was read at 580nm and the NH_4_^+^ content was estimated through the standard curve.

### Assay of the enzyme activities

The activities of glutamate synthase (GOGAT), glutamine synthetase (GS), glutamate dehydrogenase (GDH), and nitrate reductase (NR) were evaluated based on the methods of Lin and Kao (1996) and Gangwar et al. (2011). The root tissue and extraction solution of the transcriptomic analysis were homogenized in ice bath in the ratio of 1:5. After centrifugated at 4°C with 150,00×g for 20 min, the supernatant of the homogenized solution was collected as the crude solution for measuring the enzyme activities.

The GS crude enzyme solution was extracted with 50 mM Tris-HCl buffer (pH 8.0, 2 mM MgCl_2_, 2 mM DTT, 0.4 M sucrose). The mixture of 0.7 mL of the crude enzyme solution with 1.6 mL of 0.1 M Tris-HCl buffer (pH 7.5, 2 mM MgCl_2_, 20 mM sodium glutamate, 20 mM cysteine, 2 mM EGTA, 80 mM hydroxylamine hydrochloride) and 0.7 mL of 40 mM ATP was incubated at 37°C for 30 min. After addition of 1 mL of color developing agent (0.2 M TCA, 0.35 M FeCl_3_, 0.6 M HCl), the absorbance was measured at 540 nm.

The crude enzyme solution of GOGAT was extracted with 10 mM Tris-HCl buffer (pH 7.5, containing 1 mM MgCl_2_, 1 mM EDTA, 1 mM DTT). A total of 0.5 mL of crude enzyme solution was mixed with 0.05 mL of 0.1 M α-Ketoglutarate, 0.1 mL of 10 mM KCl, 0.2 mL of 3 mM NADH and 1.75 mL of buffer. After added 0.4 mL of 20 mM glutamine, the absorbance changes for 3 min were immediately recorded at 340 nm. The crude enzyme solution of GDH was extracted with 0.2 M Tris-HCl buffer (pH 8.0). One mL of the crude enzyme solution was thoroughly mixed with 0.3 mL of 0.1 M α-Ketoglutarate, 0.3 mL of 1 M NH_4_Cl, 0.2 mL of 3 mM NADH and 1.2 mL of buffer. The decrease of absorbance was recorded for 3 min at 340 nm. The crude enzyme solution for NR was extracted with 25 mM phosphate buffer (pH 8.7, 10 mM cysteine, 1.3 mM EDTA). 1.5 mL of the crude enzyme solution was mixed with 1.2 mL of 0.1 M KNO_3_ phosphoric acid buffer (pH 7.5) and 0.5 mL of 3 mM NADH and incubated in a water bath at 25°C for 1 h in dark. After added 1 mL of 1% sulphonamide solution (v/v) and 1 mL of 0.02% naphthalene ethylenediamine hydrochloride solution (v/v), the mixed reaction was kept in dark for 15 min. The absorbance was read at 540nm and the NO_2_^-^ was calculated according to the standard curve.

The activities of superoxide dismutase (SOD), peroxidase (POD) and catalase (CAT) were estimated following the methods of Decleire et al. 2012 and Maehly and Chance, 1954. The root tissues of each sample were mixed with 50 mM of PBS (pH 7.8) at a ratio of 1:10 on ice bath and centrifugated at 15000×g for 20 min at 4°C. After mixed 0.1 mL of SOD crude enzyme solution with 0.3 mL of 0.13 M methionine, 0.75 mM nitrogen blue tetrazole, 0.1 mM EDTA-Na_2_, 20 μM riboflavin and 1.7 mL PBS, the mixture solution was incubated at 4000 LX for 20 min. The absorbance was measured at 560 nm. The POD activity was evaluated by mixing 0.1 mL crude enzyme solution with 2.9 mL of 0.5% guaiacol (containing a small amount of H_2_O_2_). The increase of absorbance was recorded for 3 min at 470 nm. The mixture of 0.1 mL of the crude enzyme solution and 0.4 mL of 0.1 M H_2_O_2_ and 2.5 mL of PBS was used to measure the absorbance of CAT at 240 nm and record the changes of absorbance in 3 minutes

### Measurement of the malondialdehyde content

The content of malondialdehyde (MDA) was determined by the thiobarbituric acid method (TBA) (Li et al., 2013). The mixture of 1 g of the frozen root tissue and 10 mL of TCA was grinded for homogenization by centrifugation. Then 2 mL of the extraction solution was mixed with 0.6% TBA (v/v) and incubated in a boiling water bath for 15 min. The absorbance values were read at 532, 600, and 450 nm.

### RNA extraction, cDNA library preparation, and sequencing

Total RNA was extracted from the root tissues by using the Plant RNA Purification Reagent (Invitrogen, USA) following the manufacturer’s instructions. The potential contaminating genomic DNA was removed from RNA preparation with the DNase I kit (TaKara Bio, Japan). RNA integrity was checked by 1% agarose gel electrophoresis. Total RNA concentration was determined using a 2100 Bioanalyser (Agilent Technologies, USA) and RNA quality was assessed using the ND-2000 NanoDrop (NanoDrop Technologies, USA).

RNA-seq libraries were constructed using the TruSeq™ RNA sample preparation kit from Illumina (San Diego, USA). 1,000 ng of total RNA was used to isolate mRNA using a polyA selection method by oligo(dT) beads. The extracted mRNA was fragmented in the first-strand synthesis buffer by heating at 94°C, followed by first-strand cDNA synthesis using reverse transcriptase and random primers. Synthesis of double-stranded cDNA was performed using the 2nd strand master mix provided using a SuperScript double-stranded cDNA synthesis kit (Invitrogen, USA). Resulting double-stranded cDNA was end-repaired, dA-tailing and ligated with NEBNext adaptors. Libraries were fragmented as 300 bp on 2% Low Range Ultra Agarose. Finally, libraries were enriched by 15 cycles of amplification. After quantified by TBS380, paired-end RNA-seq libraries were sequenced with the Illumina HiSeq xten/NovaSeq 6000 sequencer (2 ×150bp read length). The data presented in the study are deposited in the NCBI repository, accession number PRJNA874354

### Bioinformatics and RNA-seq analysis

The transcriptomic analysis was based on the genome of *Zea mays* (GCF_902167145.1, https://www.ncbi.nlm.nih.gov/genome/?term=Zea_mays) from the NCBI database. The acquired paired-end reads were trimmed and quality controlled by SeqPrep (https://github.com/jstjohn/SeqPrep) and Sickle with default parameters. The clean reads in each sample were independently mapped to the reference genome with the orientation mode of the HISAT2 package (Kim et al., 2015). The mapped reads of each sample were further assembled by using the reference-based approach of the StringTie package (Pertea et al., 2015).

The differential expression of genes between the control and treatment groups were calculated based on the transcripts per million reads (TPM) by using the DESeq2 (Love et al., 2014). Genes with |log_2_^fold change^| ≥ 1 and *p-adjust* < 0.05 were considered to be significantly different. For functional enrichment analysis, significantly up- and down-regulated genes (Benjamini-Hochberg with an FDR ≤ 0.05) were selected for GO and KEGG metabolic pathways analysis by using the Goatools (https://github.com/tanghaibao/Goatools)and KOBAS (http://kobas.cbi.pku.edu.cn/home.do) (Xie et al., 2011). The outputs were generated by AI and the GraphPad Prism (v 8.0) and the significant differences were evaluated by the IBM SPSS Statistics (v 25) package.

### qRT-PCR Assays

cDNAs were synthesized from the purified 100 ng total RNA using a PrimeScript™ RT Reagent Kit and gDNA Eraser (TaKaRa, China). The quantitative real-time polymerase chain reaction (qRT-PCR) analysis was conducted with a TB Green *Premix Ex Taq*™ II Kit (Takara, China). The reaction mixture contained 12.5 μL TB Green Premix Ex Taq, 1 μL forward and reverse primers respectively, 1 μL cDNA and 9.5 μL ddH_2_O. The qPCR thermal cycling was as follows: 95 °C for 30 s followed by 40 cycles of 95 °C for 5 s, 55 °C for 30 s and 72 °C for 30 s. Each reaction was repeated three times. In every qPCR run, *ZmGAPDH* was used as an internal control to minimize systematic variations in the amount of cDNA template. The primers used for qRT-PCR were listed in **Table S1**.

## Results

### Effect of histidine on root morphology under salt stress

**Figure 1:**
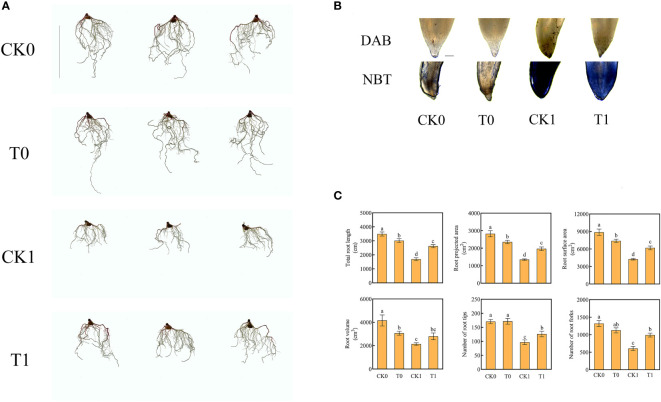
Root morphology (Ruler represents 20cm) **(A)**, root tips with DAB and NBT staining (Ruler represents 200 μm) **(B)**, total root length, root projected area, root surface area, root volume, number of root tips, number of root forks **(C)** in the four different treatments. Data were presented as the mean ± SEM for six biological replicate samples. Bars labeled with different letters indicated significant difference between treatments.

Our results confirmed that when the root was under salt stress, the total length of root (all fibrous roots) was significantly increased by adding histidine, the average length increased from 1690 cm to 2621 cm and the root projected area and surface area enlarged by 45.8% and 45.31%, respectively. In addition, the numbers of the root furcation and root tips were significantly increased by 63.2% and 30.12%, respectively (**Figure 1C**).

### Effect of histidine on the ROS accumulation under salt stress

**Figure 2:**
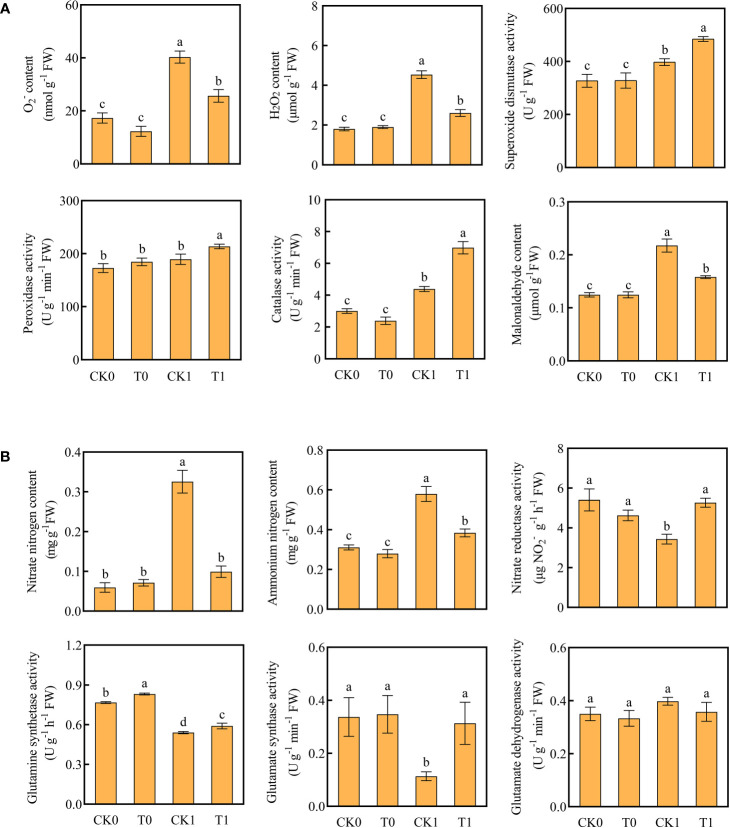
Contents of O_2_^-^, H_2_O_2_, MDA, antioxidant enzyme activities **(A)** and contents of ammonium nitrogen, nitrate nitrogen, nitrogen metabolizing enzyme activity **(B)** in the four treatments. Data are presented as the mean ± SEM for six biological replicate samples and each sample included 3 plants. Bars labeled with different letters for significant difference between treatments.

The level of ROS is related to the adaptation of plants to salt stress. After salt stress treatment, the O_2_^‐^ level of root was significantly increased. When the roots were further treated with histidine, the ROS levels were significantly increased, while the accumulative levels of O_2_^‐^ and H_2_O_2_ were significantly reduced (**Figure 2A**). The DAB and NBT staining results revealed that the root tips showed dark brown and deep blue patches with the salt stress treatment, but the patch colors became light after the histidine treatment (**Figure 1B**). Antioxidant enzymes are very important in the process of salt tolerance of plants, and they are often used as indicators to evaluate plant salt tolerance. The analysis of enzyme activities showed that, after the treatment of histidine, the activities of SOD, POD and CAT in the root tissues were significantly increased by 21.92%, 12.84% and 59%, respectively (**Figure 2A**), which were significantly higher than those with the salt stress treatment. In addition, the content of MDA was significantly reduced after the histidine treatment.

### Effects of histidine on the nitrogen use efficiency in maize with salt stress treatment

When the roots were treated with the salt stress, the contents of nitrate and ammonium nitrogen were significantly accumulated; while the activities of NR, GS, and GOGAT were significantly decreased (**Figure 2B**). However, after the histidine treatment, compared to the control group (CK1), the contents of nitrate nitrogen and ammonium nitrogen in roots were significantly decreased by 69.54% and 34.48%, respectively, while the activities of NR, GS, and GOGAT were increased significantly, by 53.2%, 9.26% and 176.52%, respectively.

### Expression profiles of transcripts

A total of 1,253,271,938 raw paired-end reads were obtained from the Illumina sequencing platform. After quality control, 1,239,263,410 clean paired-end reads with 53.86 - 54.74% of GC content were used in transcriptomic analysis. All samples were independently aligned with the reference genome using HISAT2. About 87.98% - 89.75% of the reads were mapped to the maize genome and 85.29% - 86.96% of reads uniquely mapped to the reference sequences (**Table S2**).

Based on the reference genome, the mapped reads in each sample were processed using the StringTie package and the assembled contigs were compared with the original genomic annotation information for novel transcripts and genes. A total of 52,229 transcripts with >1,800 bp and 4,432 transcripts with ≤ 200 bp were derived. The search against the known transcripts of the genome identified 28,976 potential novel transcripts, 5,977 of which may produce new genes (**Figure S2**).

**Figure 3:**
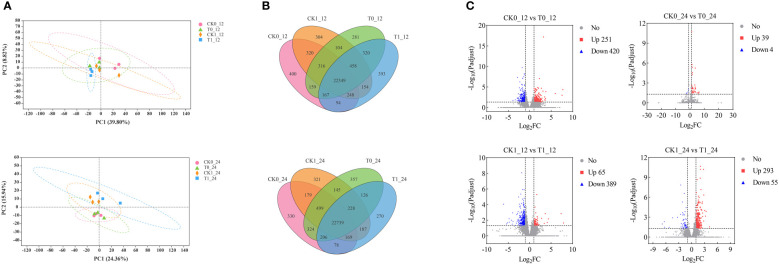
Identification and analysis of DEGs in the *Zea mays* root samples of CK0, T0, CK1, and T1. **(A)** PCA analysis of 4 treatment groups. **(B)** The Venn diagram for common and/or unique DEGs among those groups using. **(C)** Differentially expressed genes between groups.

The principal component analysis (PCA) revealed that the CK0 and T0 samples at 12 h and 24 h showed no difference along PCI (39.80% and 24.36%) and PCII (8.82% and 15.94%). The CK1 and T1 treatment samples were not significantly different along PCI, but they could be separated as two different groups along the PCII at 12h and 24h (**Figure 3A**). The unique and shared genes across all samples were identified by the Venn diagram analysis (**Figure 3B**). At 12 h and 24 h after treatment, 400, 281, 304, 393 and 330, 357, 321, and 270 uniquely expressed genes were found in the CK0, T0, CK1 and T1 samples, and 22,349 and 22,739 co-expressed genes of those samples, respectively. In the RNA-seq analysis by DESeq2, genes with *p-*adjust < 0.05, |log_2_^fold change^| ≥ 1 were defined as DEGs in our analysis. A total of 671 (up 251, down 420), 454 (up 65, down 389), 43 (up 39, down 4) and 348 (up 293, down 55) DEGs were found in the analysis of the CK0_12 vs T0_12, CK1_12 vs T1_12, CK0_24 vs T0_24 and CK1_24 vs T1_24, respectively (**Figure 3C**). The details of the DEGs were presented in **Table S3**.

### Functional annotation of genes

**Figure 4:**
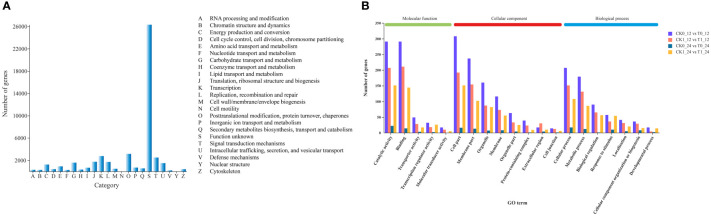
Cluster of Orthologous Groups (COG) function categories of unigenes **(A)** and the Gene Ontology (GO) classification of DEGs **(B)** in *Zea mays*.

All genes obtained from the transcriptomic assembly were searched against six major databases for gene functional annotation. In a total of 47,042 (94.47%) annotated genes, 36,571, 18,364, 42,430, 46,966, 32,196, 27,808 genes were annotated by the GO, KEGG, COG, NR, Swiss prot and Pfam databases, respectively, accounting for 77.74%, 39.04%, 90.20%, 99.84%, 68.44% and 59.11% of the total annotated genes (**Table S4**). The gene function predictions based on the COG database suggested that a total of 42,430 genes were annotated into 23 annotated categories, including the largest group (n = 26,306, 62%) of unknown, followed by the posttranslational modification, protein turnover, chapters (n = 3,145, 7.41%) and the transcription (n = 2,749, 6.48%) categories. Only 7 and 5 genes were assigned to the nuclear structure and cell motility categories, accounting for 0.016% and 0.012%, respectively (**Figure 4A**).

The GO functional annotations of DEGs assigned to the biological process, cellular component and molecular function categories were presented in **Table S5** and **Figure 4B**. In all comparisons, most of DEGs were assigned to the cellular process of the biological process, the cell part in cellular component, and the binding and/or catalytic activity in molecular function.

### Functional pathway enrichment analysis

**Figure 5:**
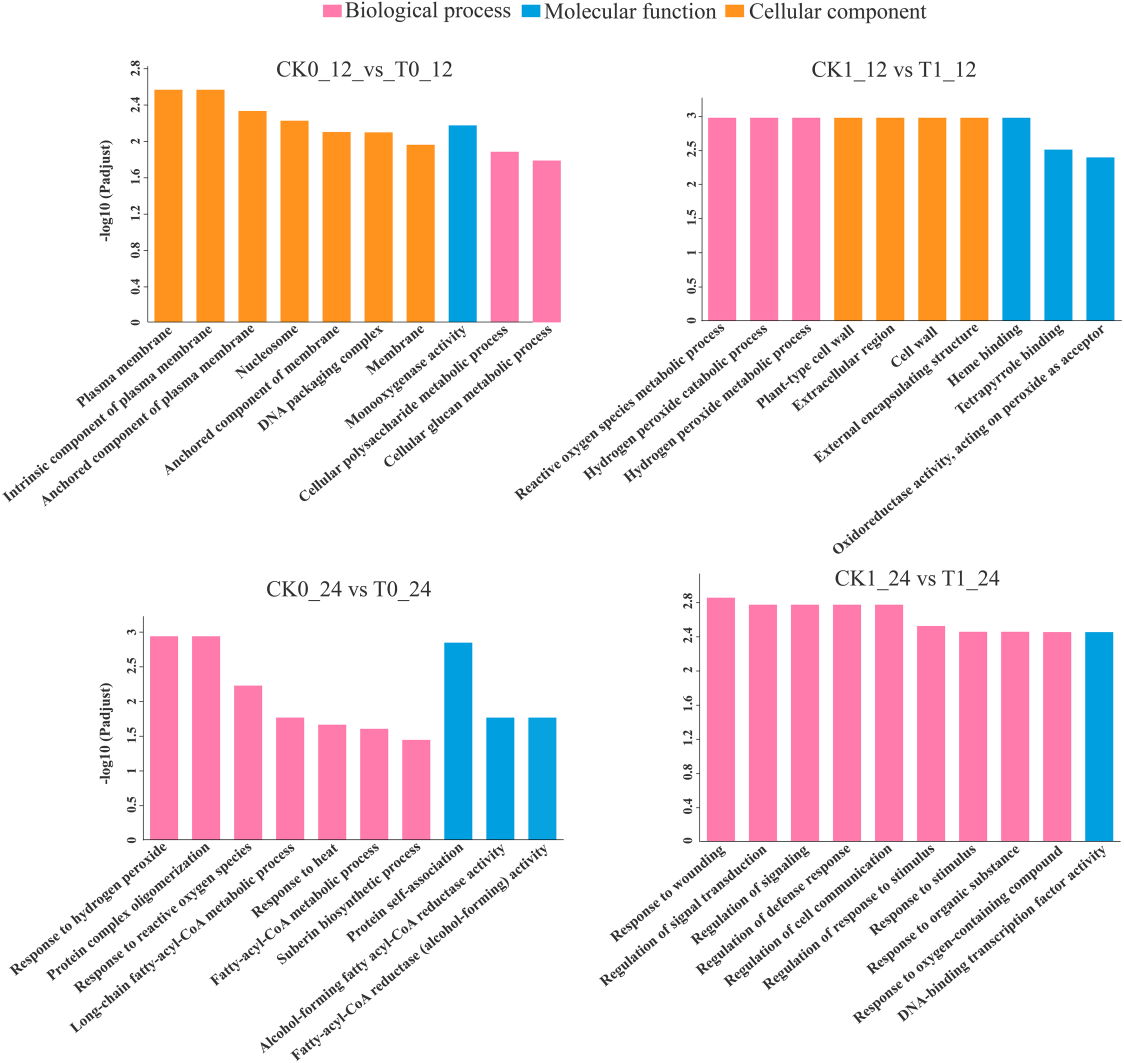
GO enrichment analysis of DEGs. *p*-adjust < 0.05 was considered to be significantly different.

The GO enrichment analysis found that the DEGs in the comparisons of the CK0_12 vs T0_12 (569 genes), CK1_12 vs T1_12 (385 genes), CK0_24 vs T0_24 (59) and CK1_24 vs T1_24 (278 genes) were enriched in 252, 146, 133, and 280 pathways, respectively (**Table S6**). With the p-adjust <0.05 as the significance threshold, about 22, 16, 12, and 32 GO pathways were significantly enriched in those analysis. Most of DEGs in the 12 h and 24 h groups with histidine treatment alone were related to the plasma membrane (GO:0005886) and response to hydrogen peroxide (GO:0042542). Under salt stress, most of DEGs in 12 h and 24 h histidine treatment groups were associated with the reactive oxygen species metabolic process (GO: 0072593) and response to wounding (GO: 0009611) (**Figure 5**)

**Figure 6:**
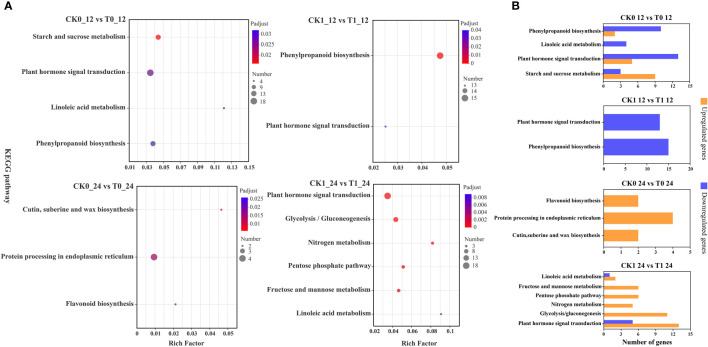
KEGG enrichment analysis of DEGs **(A)**, up- and down-regulated DEGs in KEGG pathways **(B)**. *p*-adjust < 0.05 was considered to be significantly different.

**Figure 7:**
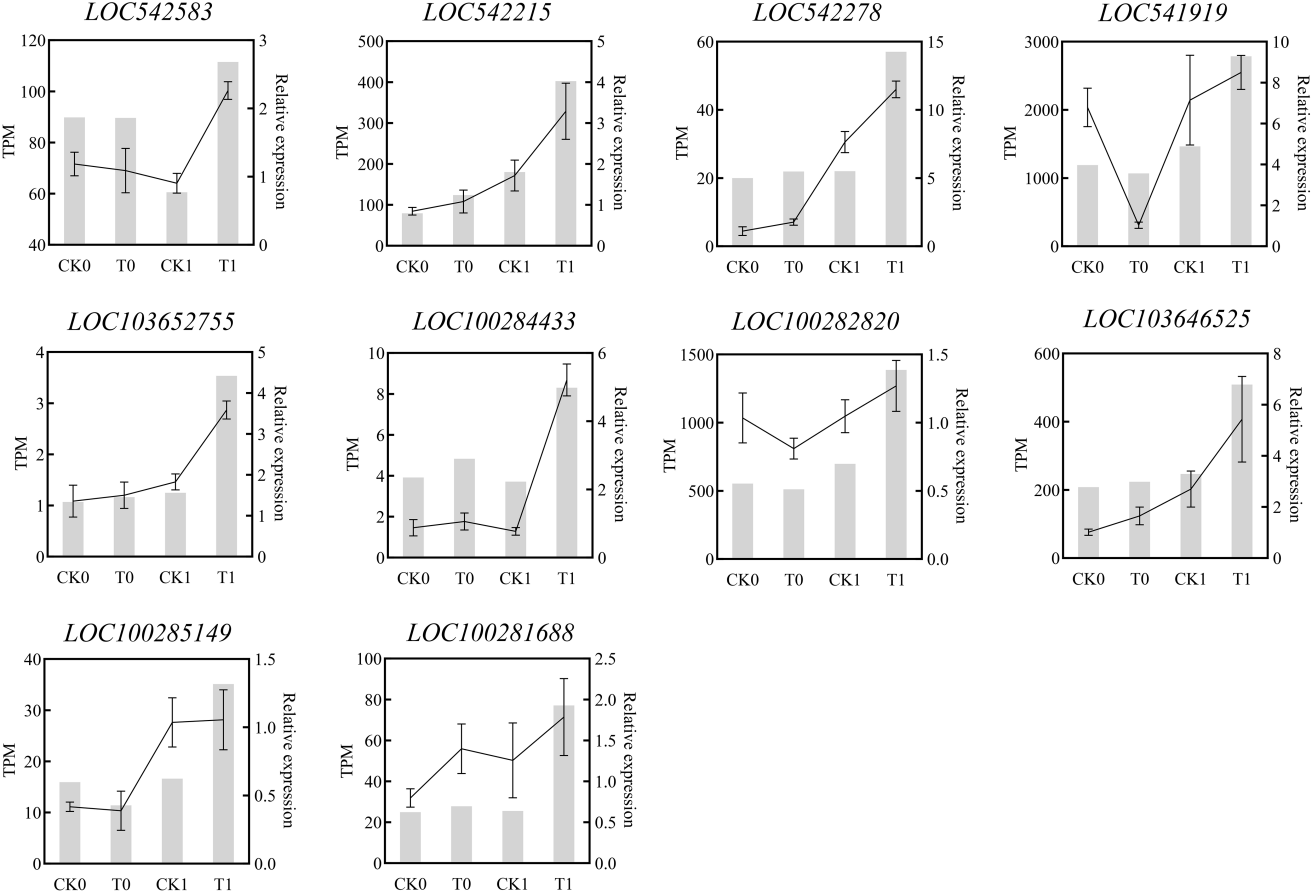
Quantitative real-time PCR (RT-qPCR) validation of the selected DEGsfrom RNA-seq analysis.The relative expression amount obtained by RT-PCR was expressed by broken lines. RT-qPCR data showed the mean values from three replicates, and the error bars represented the SEM of the means, while the corresponding expression data for RNA-seq were presented in the grey histogram.

The KEGG enrichment analysis on DEGs showed that 266, 178, 20 and 120 DEGs in the analysis of CK0_12 vs T0_12, CK1_12 vs T1_12, CK0_24 vs T0_24 and CK1_24 vs T1_24 were enriched to 76, 61, 17 and 44 pathways, respectively (**Table S7** and **Figure 6**). With the *p*-adjust < 0.05 as a cutoff, 4 enriched KEGG pathways in the analysis of CK0_12 vs T0_12 were related to the environmental information processing and starch and sucrose metabolism, plant hormone signal transduction, inoleic acid metabolism, and phenylpropanoid biosynthesis. The significantly enriched KEGG pathways in the analysis of CK0_24 vs T0_24 were related to genetic information processing and metabolism, including cutin, suberine and wax biosynthesis, protein processing in endoplasmic reticulum, flavonoid biosynthesis. Without salt stress, the DEGs in the 12 h treatment group were mainly associated with the carbohydrate metabolism, signal transmission, lipid metabolism, biosynthesis of other secondary metals. While in the 24 h groups, the DEGs were involved in lipid metabolism, biosynthesis of other secondary metals, as well as folding, sorting, and degradation. For the samples with salt stress treatment, the KEGG pathways enriched by DEGs were all associated with environmental information processing and metabolism. In the analysis of CK1_12 vs T1_12, the DEGs only in phenylpropanoid biosynthesis and plant hormone signaling transduction were significantly enriched. In the comparison of CK1_24 vs T1_24, 6 pathways related to environmental information processing and metabolism were significantly enriched, including plant hormone signal transduction, glycolysis/gluconegenesis, nitrogen metabolism, pentose phosohate pathway, fructose and mannose metabolism and linoleic acid metabolism (**Figure 6**). The RT-qPCR analysis of 10 DEGs from the enriched KEGG pathways also confirmed the consistency of the results between RNA-seq and RT-qPCR analysis (**Figure 7**).

### Integrated pathway analysis of histidine on regulating salt tolerance in maize roots

**Figure 8:**
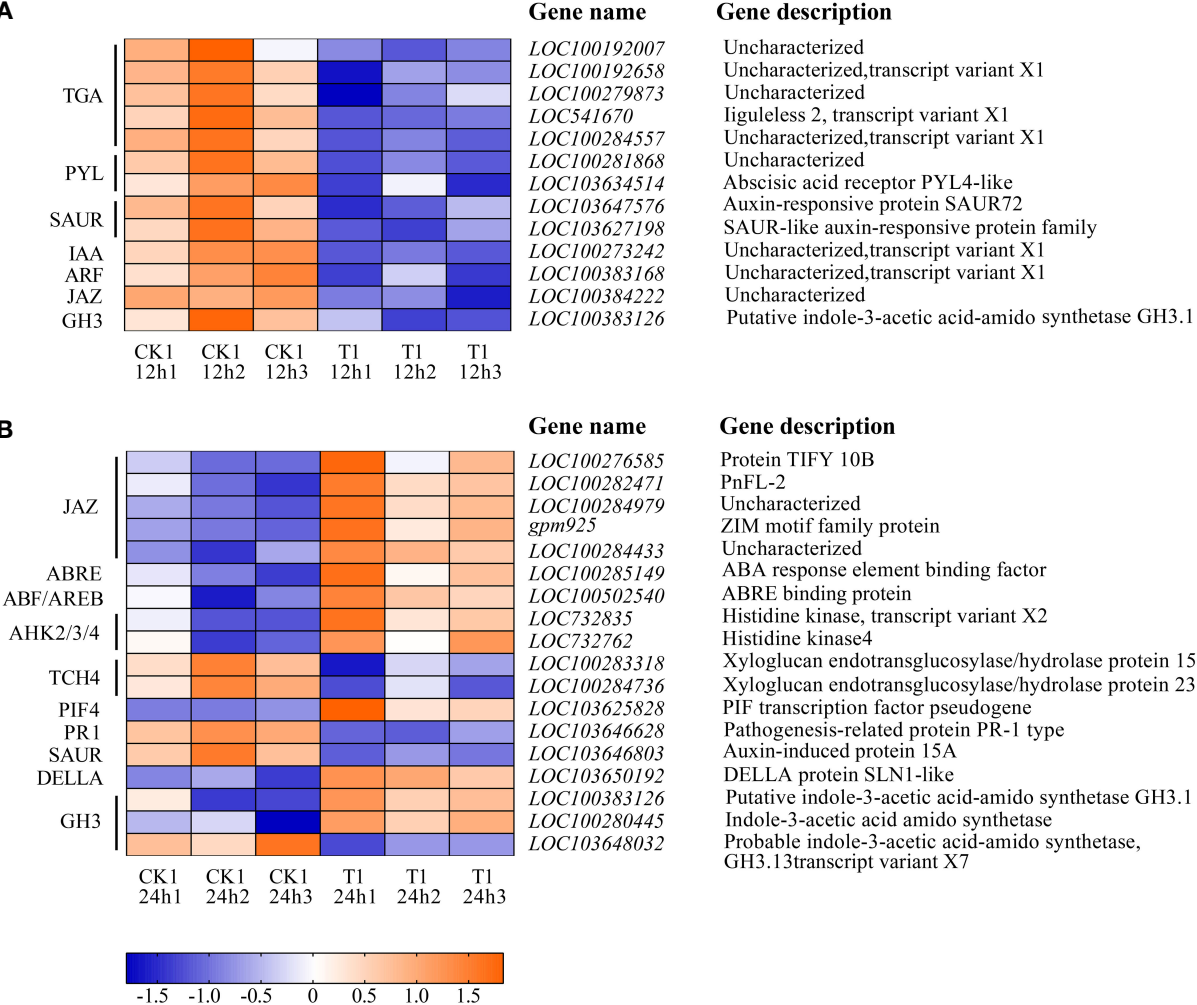
Heatmap of the DEGs involved in plant hormone signal transduction between CK1 and T1. **(A)** Treatment for 12 h. **(B)** Treatment for 24 h. The color in the figure indicates the expression magnitude of the gene after normalization treatment in each sample. Orange means upregulated expression of genes, and blue means downregulated expression of genes. The number in each sample name represents the sample order.

**Figure 8** listed the DEGs of the samples in CK1 and T1 groups that are related to the plant hormone signal transduction. After 12 h of histidine treatment, all DEGs in T1 were downregulated, including Auxin early response genes (AUX/IAA), auxin response factor (ARF), small auxin upregulated RNA(SAUR), gretchen hagen3 (GH3), Abscisic acid receptor (PYL), Jasmonic acid inhibitor (JAZ). However, after 24 h of histidine treatment, some DEGs were up-regulated and some down-regulated. Thirteen up-regulated genes were involved GH3, cytokinin receptor (AHK2/3/4), DELLA, phytochrome interaction factors 4 (PIF4), ABA response element (ABRE), ABRE binding protein (AREB/ABF), and JAZ. Five down-regulated genes were involved GH3, SAUR, and TCH4.

**Figure 9:**
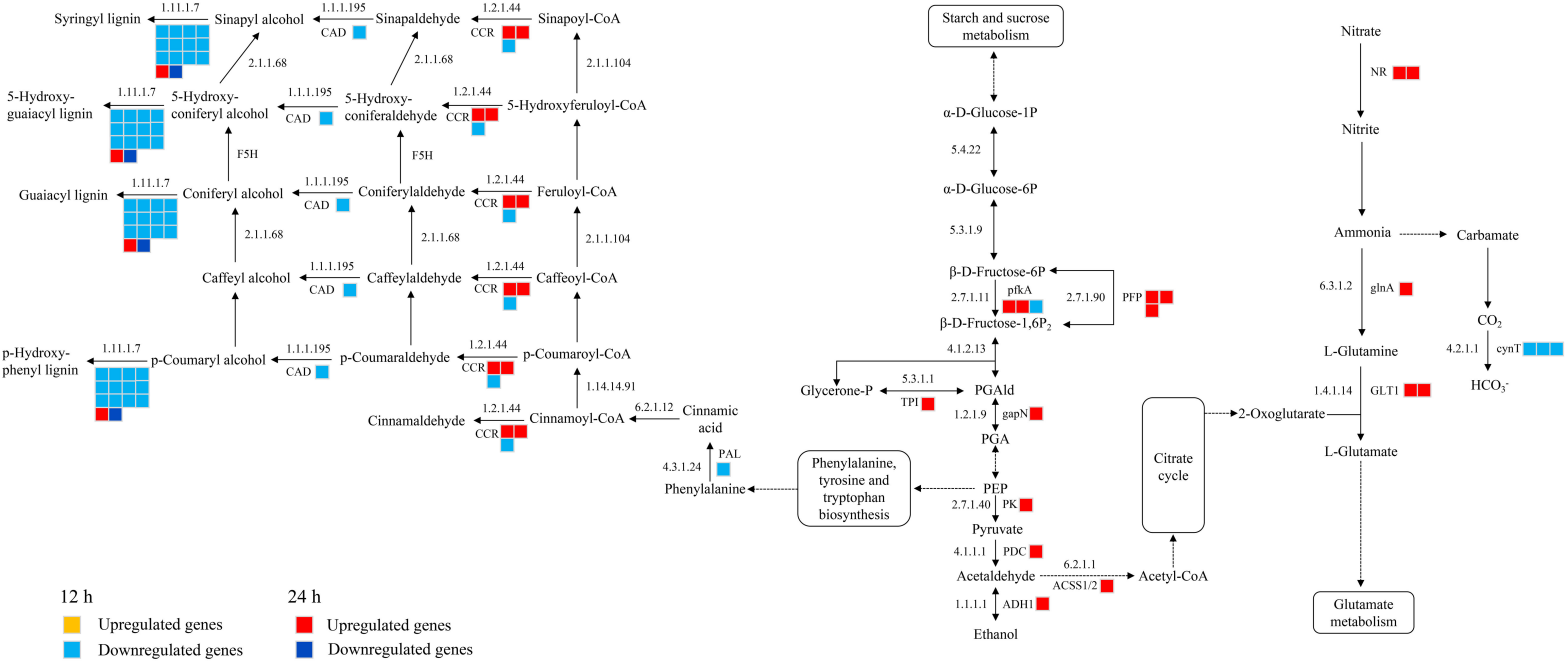
Glycolysis, nitrogen metabolism, phenylpropanoid biosynthetic metabolic pathways in histidine treatment atb12 h and 24 h under salt stress. Abbreviations are as follows: NR, nitrate reductase [NADH]; glnA, glutamine synthetase; GLT1, glutamate synthase [NADH]; cynT, carbonic anhydrase; pfkA, 6-phosphofructokinase; PFP, pyrophosphate fructose 6-phosphate 1-phosphotransferase; TPI, triose phosphate isomerase; gapN, glyceraldehyde-3-phosphate dehydrogenase; PK, pyruvate kinase; PDC, pyruvate decarboxylase; ADH1, alcohol dehydrogenase; ACSS1/2, acetyl-coenzyme A synthetase; PAL, phenylalanine ammonia lyase; CCR, cinnamoyl-CoA reductase; CAD, cinnamyl alcohol dehydrogenase; E1.11.1.7, lignin peroxidase.

A total of 39 DEGs were identified that are associated with the phenylpropanoid biosynthesis, glycolysis and nitrogen metabolism were identified in CKI and T1 groups. In the 24 h treatment group, 11 and 5 up-regulated genes were significantly enriched in glycolysis and nitrogen metabolism, respectively. In glycolysis, these up-regulated genes were related to 6-phosphofructokinase (pfkA), pyrophosphate fructose 6-phosphate 1-phosphotransferase (PFP), triose phosphate isomerase (TPI), glyceraldehyde-3-phosphate dehydrogenase (gapN), pyruvate kinase (PK), pyruvate decarboxylase (PDC), alcohol dehydrogenase (ADH1), and acetyl-coenzyme A synthetase (ACSS1/2). In nitrogen metabolism, these up-regulated genes were related to nitrate reductase [NADH] (NR), glutamine synthetase (glnA), and glutamate synthase [NADH](GLT1). In the 12 h treatment group, 1 and 3 down-regulated genes were enriched in glycolysis and nitrogen metabolism, respectively, whichis related to pfkA and carbonic anhydrase (cynT) in glycolysis and nitrogen metabolism. In the 12 h treatment group, 15 down-regulated genes were significantly enriched in the phenylpropanoid biosynthesis, phenylalanine ammonia lyase (PAL), cinnamoyl-CoA reductase (CCR), cinnamyl alcohol dehydrogenase (CAD), and peroxidase (E1.11.1.7) (**Figure 9**).

## Discussion

The interaction between plant and environment is a constant and essential activity throughout the plant life cycle, which influences plant growth, development, and survival. In arid and semi-arid regions, soil salinization is an important worldwide environmental problem. As one of the most important crops and the main material for bioethanol production in China, maize crops are extremely sensitive to drought and salt stresses, which significantly affect plant growth and development. The results of the present study showed that histidine can protect the maize root system from salt stress and enhance the abiotic stress tolerance of maize. When applied exogenously to maize exposed to salt stress, histidine not only resulted in increased growth and other physiological characteristics of roots, but also overcame the environmental salt stress by activating the antioxidant enzyme activities and regulating nitrogen metabolism and plant hormone signal transduction pathways.

Roots are highly sensitive to changes in their surrounding environment and the responses to stresses such as salinity and drought are very dynamic and complex in nature (Duan et al., 2015). Studies have shown that the addition of amino acids from hydrolyzed meat meal will induce structural changes in maize roots (Ertani et al., 2010; Colla et al. (2015). Our results confirmed that salt stress caused damage to maize roots and reduced the root area, number of lateral roots, and root length. However, after histidine was applied, the root length, root projected area, root surface area, root volume, and numbers of root tips, and furcation were significantly increased (**Figure 1**).

Plants antioxidant system plays an important role in resisting environmental stresses. Salinity exposure causes enhanced energy consumption and often enhanced respiration which are directly linked to the enhanced production of ROS (Tiwari et al., 2002). Acting as signaling molecules including hydroxyl radicals (OH), hydrogen peroxide (H_2_O_2_), and superoxide anion (O_2_^‐^), ROS can trigger signal transduction pathways in response to salt stress (Jiang et al., 2012). Previous studies suggested that plant roots could absorb amino acids, which in turn as bio-stimulants could improve the ability of plants to resist salt stress (Watson and Fowden, 1975; Colla et al., 2014). For example, proline can remove free radicals in plants, significantly increase the activity of antioxidant enzymes, and reduce the content of MDA (Ashraf and Foolad, 2007; Satoh et al., 2002; Rady et al., 2019). Exogenous ornithine and glutamate and γ-aminobutyric acid can increase the activities of CAT, SOD, POD, and reduce the Na^+^/K^+^ ratio and content of H_2_O_2_ and MDA under salt stress (Chang et al., 2010; Da Rocha et al., 2012; Kaspal et al., 2021; Ahmadi et al., 2021). Our experiments revealed that the histidine treatment significantly increased the resistance enzyme activities of SOD, CAT, and POD in maize roots and decreased the accumulation of O_2_·^‐^, H_2_O_2_, and MDA (**Figures 1A** and **2A**). In the antioxidant enzymatic system of plants, SOD forms the first line of defense against oxidative stress POD is to oxidize phenolic compounds (Hasanuzzaman et al., 2020), and CAT can rapidly decompose H_2_O_2_ to produce H_2_O and O_2_ (Hao et al., 2021). The increased CAT activity and the reduced H_2_O_2_ content in the maize roots demonstrated that histidine can mitigate oxidative damage and improve the tolerance of maize to salt stress.

Plants can adapt to salinity stress through flexible regulation of hormone levels and/or signaling. Accumulating evidence indicates that plant hormones, besides controlling plant growth and development under normal conditions, also mediate various environmental stresses (e.g., salt stress) and thus regulate plant growth adaptation. It has been proved that amino acids are closely associated with plant hormones. For example, auxins are a group of plant hormones that affect plant growth and development. The exogenous L-glutamate acid can affect the auxin level of the root tips in *Arabidopsis thaliana* and L-tryptophan can improve growth and photosynthetic capacity as the precursor of auxin synthesis (Müller et al., 1998; Walch-Liu 2006). Our results revealed that, after 24 h of the histidine treatment, the DEGs involved in 5 plant hormone signal transduction pathways were significantly up-regulated, including the growth promoting hormones in auxin (IAA), cytokinin (CTK), and gibberellin (GA) and the stress response hormones in abscisic acid (ABA), and jasmonic acid (JA) signal transduction pathways (**Figure 8**).

The plant hormone auxin controls growth and developmental responses throughout the life of a plant. The auxin early response gene Gretchen Hagen3 (*GH3*) plays dual roles in plant development and responses to biotic or abiotic stress. GH3 genes participates in auxin homeostasis by catalyzing auxin coupling and binding of free IAA with amino acids (Jain et al., 2006). GH3 can regulate the activity of indole-3-acetic acid amido synthetase, indole-3-acetic acid amido synthetase produces IAA conjugates containing various amino acids, which changes the sensitivity of seedlings to auxin. The results of this study also confirmed that histidine can induce GH3 (*LOC100280445*; *LOC100383126*) expression, which involved in IAA synthesis and regulate the content of free IAA in maize roots (Feng et al., 2015) to improve the tolerance of maize to environmental stress.

CTK is another major phytohormone that not only regulate the plant growth/development but also play an important role during stress and in the nutrient metabolic pathway of crop plants. Research showed that the decrease of CTK level will improve the survival ability of plants under salt stress (Nishiyama et al., 2011; Pizarro et al., 2021). After 24 h of the histidine treatment, genes associated with CTK signaling pathway were up-regulated, including genes in AHK2/3/4 (*LOC732762*; *LOC732835*). AHKs act as receptors during cytokinin signaling and play a significant role in providing cytokinin function during plant development (Cucinotta et al., 2020). AHKs also can regulate histidine kinase activity, and sense CTK signal and mediate the function of CTK. Previous research reported that drought, cold and salt stress can induce the expression of AHK3 and the survival rate of *ahk* mutant increased under salt stress and drought (Tran et al., 2007; Kumar and Verslues, 2015). The up-regulated DEGs related to CTK signal transduction may be closely linked to the histidine treatment, which participated in signal transduction through AHK and act as signal molecules to interact with specific receptors on the cell membrane, causing changes in plant morphology, physiology and biochemistry as other amino acids reported by Ryan and Pearce (2001).

GA is a plant hormone and controls major aspects of plant growth such as germination, elongation growth, flower development, and flowering time. DELLA proteins are negative regulators of GA signaling that act immediately downstream of the GA receptor. When the maize roots were treated with histidine, the genes related to DELLA (*LOC103650192*) and PIF4 (*LOC103625828*) in the GA signal transduction pathway were up-regulated. In the absence of GA, DELLA will block the binding of PIF3 and PIF4 to DNA, thus inhibiting the growth of hypocotyl. In the presence of GA, it will form a GA-GID1-DELLA complex, leading to the ubiquitination and degradation of DELLA, causing PIF expression and promoting hypocotyl elongation (Li et al., 2016). This suggested that DELLA proteins promote the expression of downstream negative components of GA signaling and provide a direct feedback mechanism for regulating GA homeostasis.

ABA is an important phytohormone regulating plant growth, development, stress responses to abiotic stress, and control of seed dormancy and germination. Plants will produce ABA under high salt stress, and ABA will improve the stress resistance of plants (Nakashima and Yamaguchi-Shinozaki, 2013). In the present study ABRE (*LOC100285149*) and AREB/ABF (*LOC100502540*) were up-regulated under the histidine treatment. ABA activates the expression of many genes through the ABRE in promoter region. AREB/ABF regulates the transcription of downstream target genes mediated by ABRE (Zhu, 2002). The AREB/ABF overexpressing in maize root suggested that the addition of histidine can improve the stress resistance by regulating the expression of genes related to the stress response hormones. Several other studies also reported that *AREB/ABF*-overexpressing plants showed ABA hypersensitivity and enhanced tolerance to abiotic stresses, such as freezing, drought and salt stress in *Arabidopsis* (Fujita et al., 2005; Fujita et al., 2013).

JA is a stress-related hormone and plays a crucial role in a variety of plant development and defense mechanisms. The JA signaling pathway is involved in the response and adaptation process of plants to abiotic stresses, including cold, drought, and salinity. JAZ proteins play pervasive roles in the response to biotic stress and development of plants. Jasmonate can promote the binding of JAZ proteins with SCF^COI1^ ubiquitin ligase, cause JAZ degradation, release MYC2 transcription factors, and cause jasmonic acid dependent gene expression. The up-regulated JAZ (*LOC100284433; LOC100282471*; *LOC100282471*; *LOC100276585*; *gpm925*) at 24 h after histidine treatment in T1 group indicated that the expression of JAZ genes play an important role in plant hormone signal transduction and defense response against salt stress.

In fact, JAZ protein is a key hub of crosstalk between JA and other hormone signaling pathways (such as auxin, gibberellin, and salicylic acid) (Kazan and Manners, 2012). JAZ can promote the transcription of GA response genes and bind with DELLA to interfere with the interaction between DELLA and PIF transcription factors and offset the growth inhibition produced by DELLA (Staswick, 2008). Studies have shown that amino acids can trigger related processes of plant hormones and induce the biosynthesis genes of jasmonic acid, abscisic acid, salicylic acid (Colla et al., 2014). Based on our results we speculate that histidine may act as a signal molecule to regulate genes involved in the plant hormone signal transduction pathways by mediating plant hormone levels.

The nitrogen sources of plants include organic nitrogen (e.g., amino acids) and inorganic nitrogen (e.g., nitrate nitrogen and ammonium nitrogen). Amino acids can be directly used for protein and other nitrogen-containing compounds synthesis (Rentsch et al., 2007) and transported through the vascular system for plant metabolism and development. They worked as the signal role in the process of nitrogen acquisition by roots to stimulate nitrogen metabolism and assimilation (Tegeder, 2012; Calvo et al., 2014). Assimilation of nitrate and ammonium are vital procedures for plant development and growth. The main enzymes involved in assimilation include NR, NIR, GS, GOGAT, and GDH. Our study demonstrated that the histidine treatment can enhance the activities of NR, GS, and GOGAT (**Figure 2B**). The mechanism of histidine involved in nitrogen metabolism in maize roots under salt stress may be like that of the above amino acids.

Most nitrogen sources in soil are in the form of NO_3_^-^. The primary sources of nitrogen for plants are nitrate (NO_3_^−^) and ammonium (NH_4_^+^). Nitrate is the form of nitrogen most used by plants for growth and development; while NH4^+^ is a critical intermediate in the metabolism of plants (Glass et al., 2002). As an inducible enzyme, nitrate reductase (NR)together with nitrite reductase (NIR) can reduce NO_3_^-^ to NH_4_^+^ (Privalle et al., 1989; Chen et al., 2017). Although excess NH4^+^ can result in root and shoot growth inhibition such as biomass reduction, oxidative stress with overproduction of reactive oxygen species (ROS), plants can eliminate or reduce the toxicity of NH_4_^+^ by either directly reacting with 2-oxoglutarate to produce glutamate or combining glutamate to generate glutamine under the catalysis of GS and then reacting with 2-oxoglutarate under the action of GOGAT to produce glutamine (Liu and von Wirén, 2017). Our study showed that the activities of NR, GOGAT, and GS in roots were still at high levels after 14 days of histidine treatment, implying that histidine can regulate the activity of NR, GS, and GOGAT and improve the utilization and transformation efficiency of nitrogen and glutamate anabolism in maze root system under salt stress.

Amino acids can promote nitrogen reduction and assimilation and increase the activity of NAD dependent NR, GS, GOGAT, and GDH in maize roots, as well as the expression of genes encoding nitrogen metabolism enzymes (Schiavon et al., 2008; Ertani et al., 2010; Colla et al, 2015). Phenylpropanoids are also key mediators of plants resistance towards a number of biotic and abiotic stresses. Phenylpropanoid metabolism in plants mainly includes phenylalanine metabolism and the synthesis of secondary metabolites such as lignin and flavonoids. After 24 h of the histidine treatment, the genes associated with NR (*LOC100383210*; *LOC542278*), glnA (*LOC542215*), and GLT1 (*LOC103636185*; *LOC103652755*) were significantly expressed (**Figure 9**), which regulated the activity of NADH-NR and NADH-GOGAT. Also, the histidine treatment induced the enrichment of CCR (*LOC103634692*; *LOC103634692*) and E1.11.1.7 (*LOC103638313*) in phenylpropane biosynthesis pathway (**Figure 9**), which enhance the defense response of plants (Schiavon et al., 2010), especially E1.11.1.7 (*LOC103638313*) which plays a role in the antioxidant system. These results confirmed that histidine can play a regulatory role on the stress resistance ability and growth and development of maize by modulating genes related to nitrogen metabolism. Although the gene expression in phenylpropane biosynthesis pathway was down-regulated at 12 h after the histidine treatment, the genes associated with the above pathways were up-regulated at 24 hours, which might imply that the lignin synthesis in maize root under salt stress was not dominant in the early phase of histidine treatment.

In plant, glycolysis is a common process to provide energy for aerobic and anaerobic respiration by decomposing sugars. When plants were exposed to high salinity, glycolysis and amino acid synthesis in leaves of wheat were enhanced and the levels of some amino acids and sugars increased, including proline, lysine, sucrose, and etc. (Guo et al., 2015). Other studies suggested that the intermediates in the glycolysis process and free amino acids can affect the metabolism of plants by stimulating the glycolysis process and the activities of glucose phosphate isomerase and pyruvate kinase ( (Fernie et al., 2004; Sukumar et al., 2017; Palumbo et al., 2018). The present study revealed that when the roots under salt stress were treated with histidine, some genes associated with glycolysis were significantly enriched, which induced the high expression levels of pfkA (*LOC100192478*; *LOC100281688*), PFP (LOC103652493; LOC101027252; LOC103632535), TPI (LOC100282142), gapN (*LOC100282142*), PK (*LOC100282142*), PDC (*LOC541919*), ADH1 (*LOC542364*), and ACSS1/2 (*LOC103646525*) (**Figure 9**). The protein hydrolysate with amino acids can change the gene expression levels in the glycolysis, tricarboxylic acid cycle and pentose phosphate pathways of maize roots (Ebinezer et al., 2020). Our findings indicated that the histidine treatment dramatically increased the glycolysis process of maize root and enhanced the salt tolerance of roots mainly through increasing glycolysis and energy consumption. This suggested that the modulation of energy metabolism is essential for a response to salinity to balance the production of ROS with the requirements for defense.

## Conclusion

In summary, histidine has been implicated in the mechanism regulating salt tolerance in plants. The results of the present study confirmed that histidine can ameliorate the adverse effects of salt stress on maize root growth. When the roots were treated with histidine for 12 h or 24 h, the activity of SOD, POD, and CAT were significantly increased, alleviating the accumulation of ROS and improving the salt tolerance of maize roots. Also, the histidine treatment enhanced the activities of NR, GS, and GOGAT and promoted the nitrogen utilization, glutamate anabolism and other amino acid anabolism in maze by expressing the genes related to nitrogen metabolism. Interestingly, transcriptomic analysis revealed that the number of upregulated DEGs and enriched pathways of the roots under salt stress were increased after 24 h of histidine treatment. KEGG enrichment analysis found that these DEGs that involved in glycolysis and plant hormone signal transduction pathways were significantly up-regulated, including the growth promoting (IAA), (CTK), and (GA) and the stress response hormones in (ABA), and (JA) signal transduction pathways. Based on the above results, we speculate that histidine may act as a signal molecule to regulate genes involved in the plant hormone synthesis, signal transduction, stress perception and metabolite production for alleviation of salt stress in the maize root system.

## Data availability statement

The data presented in the study are deposited in the NCBI repository, accession number PRJNA874354.

## Author contributions

GY and XZ designed the experiments and edited the manuscript. HJ conducted the experiments. QZ, YQ, KW and TS analyzed the data. HJ wrote the paper and prepared the manuscript. All authors contributed to the paper and approved the submitted version.

## Funding

This research was supported by the National Natural Science Foundation of China (32060424) and Talent Project of the National Ethnic Affairs Commission of China (2016).

## Conflict of interest

The authors declare that the research was conducted in the absence of any commercial or financial relationships that could be construed as a potential conflict of interest.

## Publisher’s note

All claims expressed in this article are solely those of the authors and do not necessarily represent those of their affiliated organizations, or those of the publisher, the editors and the reviewers. Any product that may be evaluated in this article, or claim that may be made by its manufacturer, is not guaranteed or endorsed by the publisher.
