# Supplementary material for: Regulation of salt tolerance in the roots of Zea mays by L-histidine through transcriptome analysis
Source: Front Plant Sci. 2022 Nov 28;13:1049954. doi: 10.3389/fpls.2022.1049954 (PMC9742451; doi:10.3389/fpls.2022.1049954)
Supplement: Supplementary file 1 [file Table_1.doc]

| **Gene** | **Forward primer sequence** | **Reverse primer sequence** |
| --- | --- | --- |
| *GAPDH* | Agc aca gcg aca tca cac tc | Aac aac ctt ctt ggc acc ac |
| *LOC542278* | CAA GGT GTG GTA CGT CAT CG | GAG ATG GCG AAC TGG ATC AT |
| *LOC542215* | Cta ttc ggc cac aag aga gc | Acg att gcc ctg tac ctg ac |
| *LOC103652755* | GCT TGA GAG AAT GGC TCA CC | GTA GCT CAA ACC CAG CAT CC |
| *LOC100281688* | AGG ATC ACC GAG AAG CAG AA | TGG ACG GGT TCC TGA CTA AG |
| *LOC542583* | CCT GAT CAG TGA TGC AAT GG | ACC CAA TGC CAC TGT CTT TC |
| *LOC100282820* | TTG CTT GCA GGT TAT GCT TG | GTT GCT AAC CTT GGC GAG TC |
| *LOC103646525* | AGC CAT GGA CAT TGA AGG AC | ACC GAC CGT GTC TAG TTT GG |
| *LOC100284433*  *LOC100285149*  *LOC541919* | GTG CTG AGC CAG TAC GTC AA  ACA ACA CCG CTC CCT GTA TC  CGA TGT GAG CAG AAC AGC AT | AAG TCC TCG AAC ACC ACC AC  AGA CGC TTC CTC TGT TTG GA  GGC AAG CAT TAG CCA AAG AG |
