# Supplementary material for: Regulation of salt tolerance in the roots of Zea mays by L-histidine through transcriptome analysis
Source: Front Plant Sci. 2022 Nov 28;13:1049954. doi: 10.3389/fpls.2022.1049954 (PMC9742451; doi:10.3389/fpls.2022.1049954)
Supplement: Supplementary file 2 [file Table_2.doc]

**Table S2 | Statistics of different processing sequencing data**

| Sample | Raw reads | Clean reads | Total mapped | Multiple mapped | Uniquely mapped | Q20 (%) | Q30 (%) | GC content (%) |
| --- | --- | --- | --- | --- | --- | --- | --- | --- |
| CK1_24h3 | 56910188 | 56354034 | 50556704(89.71%) | 1699016(3.01%) | 48857688(86.7%) | 97.8 | 93.84 | 54.37 |
| CK1_24h2 | 54729808 | 54211514 | 48407456(89.29%) | 1596751(2.95%) | 46810705(86.35%) | 97.79 | 93.85 | 54.38 |
| CK1_24h1 | 58137894 | 57593938 | 51682372(89.74%) | 1763025(3.06%) | 49919347(86.67%) | 97.82 | 93.9 | 53.86 |
| T1_24h3 | 53392190 | 52903690 | 47266451(89.34%) | 1506943(2.85%) | 45759508(86.5%) | 97.76 | 93.75 | 54.71 |
| T1_24h2 | 48368012 | 47823228 | 42591421(89.06%) | 1359717(2.84%) | 41231704(86.22%) | 97.49 | 93.17 | 54.6 |
| T1_24h1 | 56295902 | 55712396 | 49396732(88.66%) | 1624240(2.92%) | 47772492(85.75%) | 97.65 | 93.51 | 54.72 |
| T0_24h3 | 49880572 | 49407618 | 43995381(89.05%) | 1478252(2.99%) | 42517129(86.05%) | 97.78 | 93.83 | 54.37 |
| T0_24h2 | 55338572 | 54763808 | 48652092(88.84%) | 1664727(3.04%) | 46987365(85.8%) | 97.64 | 93.53 | 54.17 |
| T0_24h1 | 51747116 | 51229274 | 45836737(89.47%) | 1564857(3.05%) | 44271880(86.42%) | 97.71 | 93.6 | 54.15 |
| CK0_24h3 | 50423562 | 49865530 | 44656677(89.55%) | 1536601(3.08%) | 43120076(86.47%) | 97.63 | 93.46 | 53.89 |
| CK0_24h2 | 52640038 | 52108716 | 46439654(89.12%) | 1662426(3.19%) | 44777228(85.93%) | 97.82 | 93.96 | 54.01 |
| CK0_24h1 | 53752116 | 53198500 | 47804216(89.86%) | 1623365(3.05%) | 46180851(86.81%) | 97.81 | 93.83 | 54.22 |
| CK1_12h3 | 54886978 | 54286268 | 48720024(89.75%) | 1514611  (2.79%) | 47205413(86.96%) | 97.63 | 93.47 | 54.74 |
| CK1_12h2 | 47579108 | 46965766 | 41403641(88.16%) | 1297757(2.76%) | 40105884(85.39%) | 97.1 | 92.25 | 54.38 |
| CK1_12h1 | 50272800 | 49671044 | 44307303(89.2%) | 1373405(2.77%) | 42933898(86.44%) | 97.13 | 92.27 | 54.31 |
| T1_12h3 | 48213962 | 47617690 | 42603720(89.47%) | 1310943(2.75%) | 41292777(86.72%) | 97.23 | 92.5 | 54.2 |
| T1_12h2 | 54109502 | 53445064 | 47928355(89.68%) | 1522228(2.85%) | 46406127(86.83%) | 97.25 | 92.56 | 54.09 |
| T1_12h1 | 50941492 | 50224242 | 44667619(88.94%) | 1406211(2.8%) | 43261408(86.14%) | 96.99 | 92.01 | 54.38 |
| T0_12h3 | 48053090 | 47443196 | 42102377(88.74%) | 1352371(2.85%) | 40750006(85.89%) | 97.19 | 92.44 | 54.3 |
| T0_12h2 | 55761156 | 55083714 | 49081267(89.1%) | 1568782(2.85%) | 47512485(86.26%) | 97.32 | 92.7 | 54.42 |
| T0_12h1 | 53744160 | 53145594 | 47672409(89.7%) | 1545718(2.91%) | 46126691(86.79%) | 97.39 | 92.85 | 53.9 |
| CK0_12h3 | 54514794 | 53865530 | 47877989(88.88%) | 1467873(2.73%) | 46410116(86.16%) | 97.46 | 93.02 | 54.44 |
| CK0_12h2 | 49285626 | 48635064 | 43391820(89.22%) | 1303274(2.68%) | 42088546(86.54%) | 97.25 | 92.6 | 54.63 |
| CK0_12h1 | 44293300 | 43707992 | 38454095(87.98%) | 1177357(2.69%) | 37276738(85.29%) | 97.1 | 92.23 | 54.73 |

Total reads: Clean reads;

Total mapped: The number of clean reads that can be located on the genome;

Multiple mapped: The number of clean reads with multiple alignment positions on the reference sequence;

Unique mapped: The number of clean reads with a unique alignment position on the reference sequence
