# Supplementary material for: Regulation of salt tolerance in the roots of Zea mays by L-histidine through transcriptome analysis
Source: Front Plant Sci. 2022 Nov 28;13:1049954. doi: 10.3389/fpls.2022.1049954 (PMC9742451; doi:10.3389/fpls.2022.1049954)
Supplement: Supplementary file 4 [file Table_4.doc]

**Table S4 | Annotation of unigenes in different databases**

| Database | All unigene number | Percentage of annotated  Unigenes (%) |
| --- | --- | --- |
| GO | 31891 | 78.05 |
| KEGG | 16534 | 40.46 |
| COG | 38098 | 93.24 |
| NR | 40835 | 99.93 |
| Swiss-Prot | 29426 | 72.01 |
| Pfam | 26125 | 63.93 |
| Total_anno | 40862 | 100 |
| Total | 41594 |  |

Total_anno:Total number of genes annotated to the database.

Total:Number of total genes.
