# Supplementary material for: Regulation of salt tolerance in the roots of Zea mays by L-histidine through transcriptome analysis
Source: Front Plant Sci. 2022 Nov 28;13:1049954. doi: 10.3389/fpls.2022.1049954 (PMC9742451; doi:10.3389/fpls.2022.1049954)
Supplement: Supplementary file 5 [file Table_5.doc]

**TABLE S5 |** DEGs GO functional annotation

CK0_12 vs T0_12

| **GO ID** | **Description** | **Term Type** | **CK0_12 vs T0_12 num** | **CK0_12 vs T0_12 percent** |
| --- | --- | --- | --- | --- |
| GO:0032501 | multicellular organismal process | Biological process | 13 | 13/671 |
| GO:0065007 | biological regulation | Biological process | 90 | 90/671 |
| GO:0051704 | multi-organism process | Biological process | 13 | 13/671 |
| GO:0022414 | reproductive process | Biological process | 8 | 8/671 |
| GO:0040011 | locomotion | Biological process | 1 | 1/671 |
| GO:0050896 | response to stimulus | Biological process | 57 | 57/671 |
| GO:0000003 | reproduction | Biological process | 2 | 2/671 |
| GO:0009987 | cellular process | Biological process | 207 | 207/671 |
| GO:0051179 | localization | Biological process | 41 | 41/671 |
| GO:0032502 | developmental process | Biological process | 17 | 17/671 |
| GO:0071840 | cellular component organization  or biogenesis | Biological process | 36 | 36/671 |
| GO:0019740 | nitrogen utilization | Biological process | 1 | 1/671 |
| GO:0008152 | metabolic process | Biological process | 179 | 179/671 |
| GO:0040007 | growth | Biological process | 1 | 1/671 |
| GO:0044425 | membrane part | Cellular component | 237 | 237/671 |
| GO:0005623 | cell | Cellular component | 5 | 5/671 |
| GO:0044217 | other organism part | Cellular component | 4 | 4/671 |
| GO:0044422 | organelle part | Cellular component | 63 | 63/671 |
| GO:0032991 | protein-containing complex | Cellular component | 39 | 39/671 |
| GO:0044464 | cell part | Cellular component | 308 | 308/671 |
| GO:0016020 | membrane | Cellular component | 116 | 116/671 |
| GO:0043226 | organelle | Cellular component | 160 | 160/671 |
| GO:0009295 | nucleoid | Cellular component | 1 | 1/671 |
| GO:0099080 | supramolecular complex | Cellular component | 3 | 3/671 |
| GO:0031974 | membrane-enclosed lumen | Cellular component | 3 | 3/671 |
| GO:0005576 | extracellular region | Cellular component | 17 | 17/671 |
| GO:0044421 | extracellular region part | Cellular component | 1 | 1/671 |
| GO:0030054 | cell junction | Cellular component | 14 | 14/671 |
| GO:0090729 | toxin activity | Molecular function | 1 | 1/671 |
| GO:0005215 | transporter activity | Molecular function | 49 | 49/671 |
| GO:0005488 | binding | Molecular function | 291 | 291/671 |
| GO:0003824 | catalytic activity | Molecular function | 291 | 291/671 |
| GO:0005198 | structural molecule activity | Molecular function | 10 | 10/671 |
| GO:0098772 | molecular function regulator | Molecular function | 8 | 8/671 |
| GO:0045735 | nutrient reservoir activity | Molecular function | 1 | 1/671 |
| GO:0016209 | antioxidant activity | Molecular function | 7 | 7/671 |
| GO:0045182 | translation regulator activity | Molecular function | 4 | 4/671 |
| GO:0060089 | molecular transducer activity | Molecular function | 17 | 17/671 |
| GO:0140110 | Transcription regulator activity | Molecular function | 32 | 32/671 |
| GO:0140299 | Small molecule sensor activity | Molecular function | 2 | 2/671 |
| GO:0044183 | Protein folding chaperone | Molecular function | 2 | 2/671 |

CK1_12 vs T1_12

| **GO ID** | **Description** | **Term Type** | **CK1_12 vs T1_12 num** | **CK1_12 vs T1_12 percent** |
| --- | --- | --- | --- | --- |
| GO:0032501 | Multicellular organismal process | Biological process | 3 | 3/454 |
| GO:0065007 | Biological regulation | Biological process | 65 | 65/454 |
| GO:0051704 | Multi-organism process | Biological process | 4 | 4/454 |
| GO:0048511 | Rhythmic process | Biological process | 2 | 2/454 |
| GO:0022414 | Reproductive process | Biological process | 5 | 5/454 |
| GO:0050896 | Response to stimulus | Biological process | 36 | 36/454 |
| GO:0002376 | Immune system process | Biological process | 2 | 2/454 |
| GO:0000003 | Reproduction | Biological process | 1 | 1/454 |
| GO:0098754 | Detoxification | Biological process | 1 | 1/454 |
| GO:0009987 | Cellular process | Biological process | 151 | 151/454 |
| GO:0015976 | Carbon utilization | Biological process | 3 | 3/454 |
| GO:0051179 | Localization | Biological process | 31 | 31/454 |
| GO:0032502 | Developmental process | Biological process | 4 | 4/454 |
| GO:0071840 | Cellular component organization  or biogenesis | Biological process | 29 | 29/454 |
| GO:0008152 | Metabolic process | Biological process | 131 | 131/454 |
| GO:0040007 | Growth | Biological process | 1 | 1/454 |
| GO:0044425 | Membrane part | Cellular component | 154 | 154/454 |
| GO:0005623 | Cell | Cellular component | 2 | 2/454 |
| GO:0044217 | Other organism part | Cellular component | 1 | 1/454 |
| GO:0044422 | Organelle part | Cellular component | 33 | 33/454 |
| GO:0032991 | Protein-containing complex | Cellular component | 23 | 23/454 |
| GO:0044464 | Cell part | Cellular component | 192 | 192/454 |
| GO:0016020 | Membrane | Cellular component | 73 | 73/454 |
| GO:0043226 | Organelle | Cellular component | 87 | 87/454 |
| GO:0099080 | Supramolecular complex | Cellular component | 4 | 4/454 |
| GO:0031974 | Membrane-enclosed lumen | Cellular component | 2 | 2/454 |
| GO:0005576 | Extracellular region | Cellular component | 30 | 30/454 |
| GO:0044421 | Extracellular region part | Cellular component | 3 | 3/454 |
| GO:0030054 | Cell junction | Cellular component | 12 | 12/454 |
| GO:0090729 | Toxin activity | Molecular function | 1 | 1/454 |
| GO:0005215 | Transporter activity | Molecular function | 28 | 28/454 |
| GO:0005488 | Binding | Molecular function | 211 | 211/454 |
| GO:0003824 | Catalytic activity | Molecular function | 207 | 207/454 |
| GO:0140104 | Molecular carrier activity | Molecular function | 1 | 1/454 |
| GO:0005198 | Structural molecule activity | Molecular function | 2 | 2/454 |
| GO:0098772 | Molecular function regulator | Molecular function | 8 | 8/454 |
| GO:0045735 | Nutrient reservoir activity | Molecular function | 1 | 1/454 |
| GO:0016209 | Antioxidant activity | Molecular function | 13 | 13/454 |
| GO:0045182 | Translation regulator activity | Molecular function | 2 | 2/454 |
| GO:0060089 | Molecular transducer activity | Molecular function | 10 | 10/454 |
| GO:0140110 | Transcription regulator activity | Molecular function | 18 | 18/454 |
| GO:0044183 | Protein folding chaperone | Molecular function | 1 | 1/454 |

| **GO ID** | **Description** | **Term Type** | **CK0_24 vs T0_24 num** | **CK0_24 vs T0_24 percent** |
| --- | --- | --- | --- | --- |
| GO:0065007 | Biological regulation | Biological process | 1 | 1/43 |
| GO:0051704 | Multi-organism process | Biological process | 2 | 2/43 |
| GO:0050896 | Response to stimulus | Biological process | 10 | 10/43 |
| GO:0002376 | Immune system process | Biological process | 1 | 1/43 |
| GO:0009987 | Cellular process | Biological process | 17 | 17/43 |
| GO:0051179 | Localization | Biological process | 5 | 5/43 |
| GO:0032502 | Developmental process | Biological process | 2 | 2/43 |
| GO:0071840 | Cellular component organization  or biogenesis | Biological process | 8 | 8/43 |
| GO:0008152 | Metabolic process | Biological process | 12 | 12/43 |
| GO:0044425 | Membrane part | Cellular component | 13 | 13/43 |
| GO:0044422 | Organelle part | Cellular component | 4 | 4/43 |
| GO:0032991 | Protein-containing complex | Cellular component | 1 | 1/43 |
| GO:0044464 | Cell part | Cellular component | 16 | 16/43 |
| GO:0016020 | Membrane | Cellular component | 8 | 8/43 |
| GO:0043226 | Organelle | Cellular component | 7 | 7/43 |
| GO:0005576 | Extracellular region | Cellular component | 4 | 4/43 |
| GO:0030054 | Cell junction | Cellular component | 1 | 1/43 |
| GO:0005215 | Transporter activity | Molecular function | 3 | 3/43 |
| GO:0005488 | Binding | Molecular function | 14 | 14/43 |
| GO:0003824 | Catalytic activity | Molecular function | 22 | 22/43 |
| GO:0016209 | Antioxidant activity | Molecular function | 1 | 1/43 |
| GO:0140110 | Transcription regulator activity | Molecular function | 2 | 2/43 |

**CK0_24 vs T0_24**

CK1_24 vs T1_24

| **GO ID** | **Description** | **Term Type** | **CK1_24 vs T1_24 num** | **CK1_24 vs T1_24 percent** |
| --- | --- | --- | --- | --- |
| GO:0002376 | Immune system process | Biological process | 1 | 1/348 |
| GO:0065007 | Biological regulation | Biological process | 56 | 56/348 |
| GO:0008152 | Metabolic process | Biological process | 86 | 86/348 |
| GO:0051704 | Multi-organism process | Biological process | 10 | 10/348 |
| GO:0022414 | Reproductive process | Biological process | 8 | 8/348 |
| GO:0071840 | Cellular component organization or biogenesis | Biological process | 15 | 15/348 |
| GO:0009987 | Cellular process | Biological process | 108 | 108/348 |
| GO:0032502 | Developmental process | Biological process | 14 | 14/348 |
| GO:0032501 | Multicellular organismal process | Biological process | 10 | 10/348 |
| GO:0040007 | Growth | Biological process | 3 | 3/348 |
| GO:0048511 | Rhythmic process | Biological process | 1 | 1/348 |
| GO:0051179 | Localization | Biological process | 20 | 20/348 |
| GO:0098754 | Detoxification | Biological process | 2 | 2/348 |
| GO:0019740 | Nitrogen utilization | Biological process | 2 | 2/348 |
| GO:0050896 | Response to stimulus | Biological process | 54 | 54/348 |
| GO:0032991 | Protein-containing complex | Cellular component | 9 | 9/348 |
| GO:0005623 | Cell | Cellular component | 3 | 3/348 |
| GO:0044425 | Membrane part | Cellular component | 102 | 102/348 |
| GO:0044421 | Extracellular region part | Cellular component | 2 | 2/348 |
| GO:0044422 | Organelle part | Cellular component | 25 | 25/348 |
| GO:0043226 | Organelle | Cellular component | 82 | 82/348 |
| GO:0016020 | Membrane | Cellular component | 55 | 55/348 |
| GO:0030054 | Cell junction | Cellular component | 6 | 6/348 |
| GO:0005576 | Extracellular region | Cellular component | 10 | 10/348 |
| GO:0044217 | Other organism part | Cellular component | 2 | 2/348 |
| GO:0044464 | Cell part | Cellular component | 151 | 151/348 |
| GO:0045182 | Translation regulator activity | Molecular function | 1 | 1/348 |
| GO:0140110 | Transcription regulator activity | Molecular function | 26 | 26/348 |
| GO:0016209 | Antioxidant activity | Molecular function | 2 | 2/348 |
| GO:0005215 | Transporter activity | Molecular function | 17 | 17/348 |
| GO:0098772 | Molecular function regulator | Molecular function | 4 | 4/348 |
| GO:0140299 | Small molecule sensor activity | Molecular function | 2 | 2/348 |
| GO:0005488 | Binding | Molecular function | 144 | 144/348 |
| GO:0060089 | Molecular transducer activity | Molecular function | 5 | 5/348 |
| GO:0003824 | Catalytic activity | Molecular function | 151 | 151/348 |
