# Supplementary figures and images for: Regulation of salt tolerance in the roots of Zea mays by L-histidine through transcriptome analysis
Source: Front Plant Sci. 2022 Nov 28;13:1049954. doi: 10.3389/fpls.2022.1049954 (PMC9742451; doi:10.3389/fpls.2022.1049954)

A

CK0

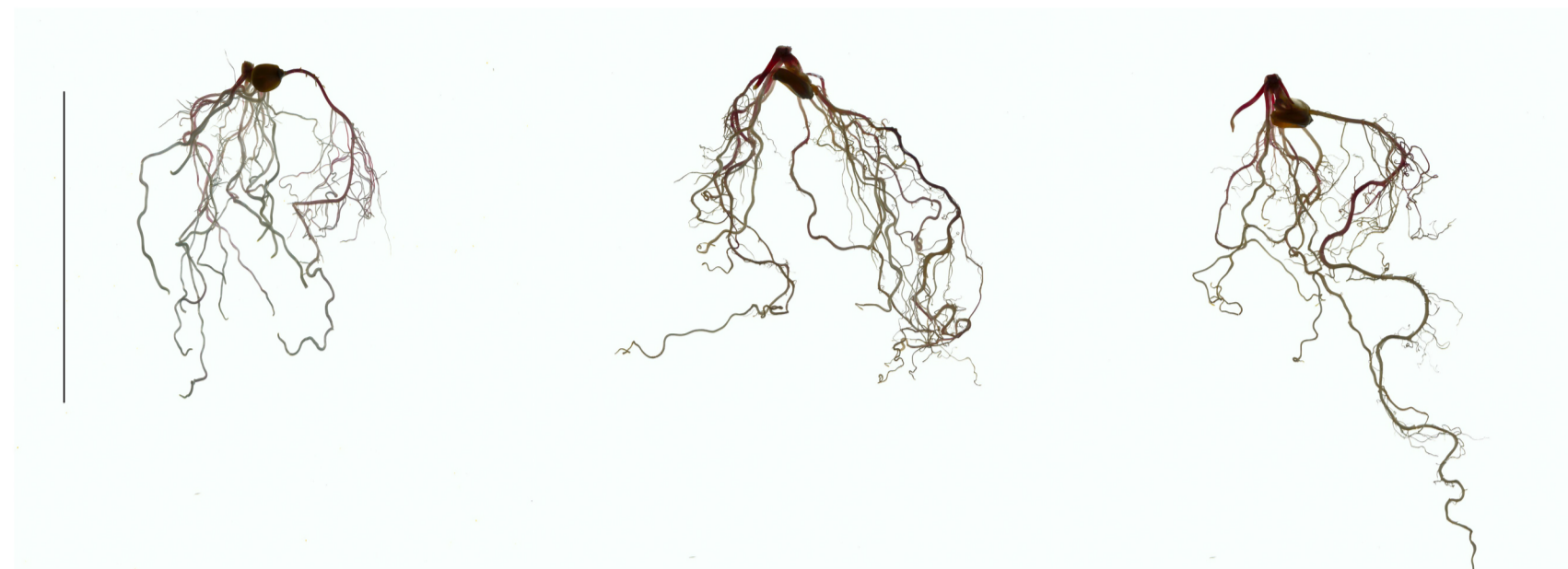

CK1

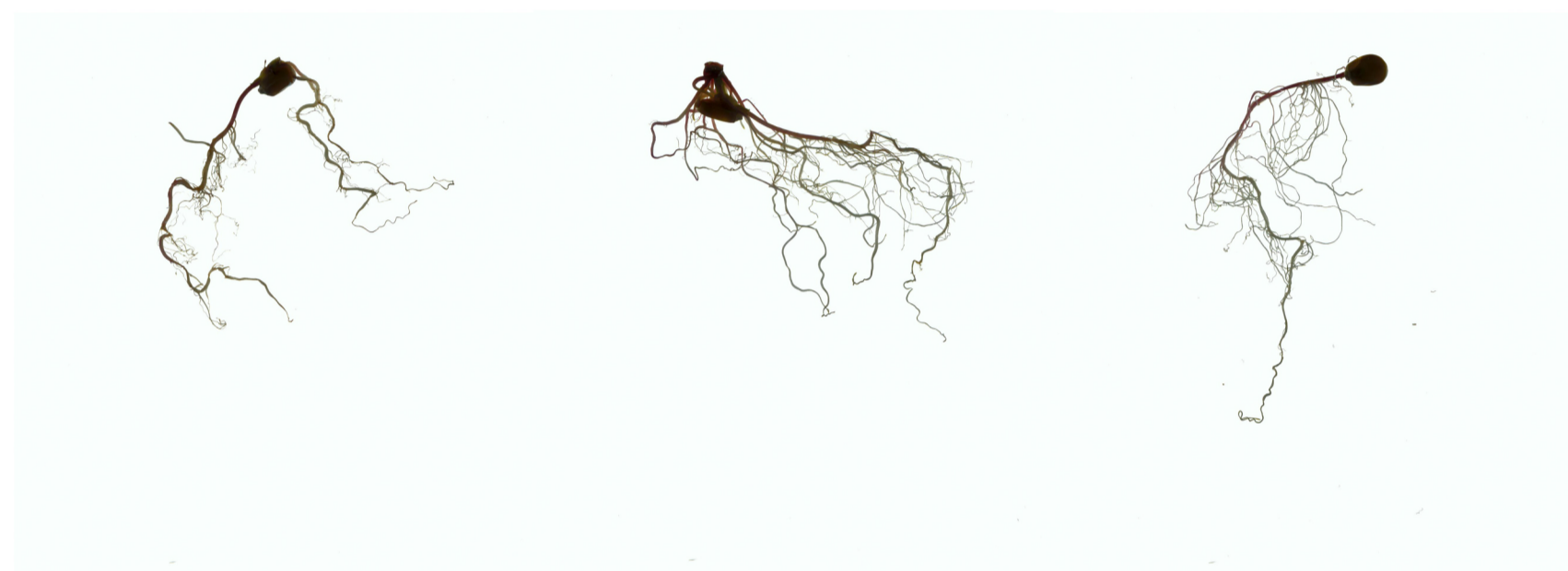CK1+100  $\mu\text{M}$ 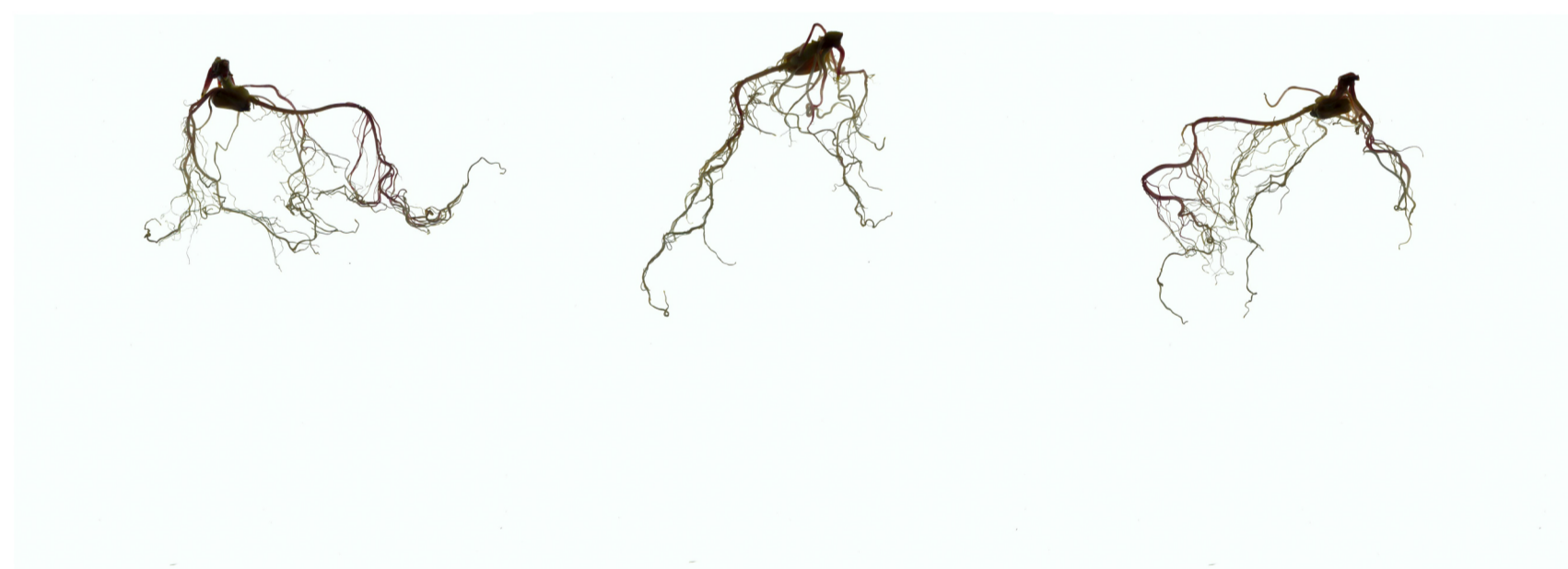CK1+10  $\mu\text{M}$ 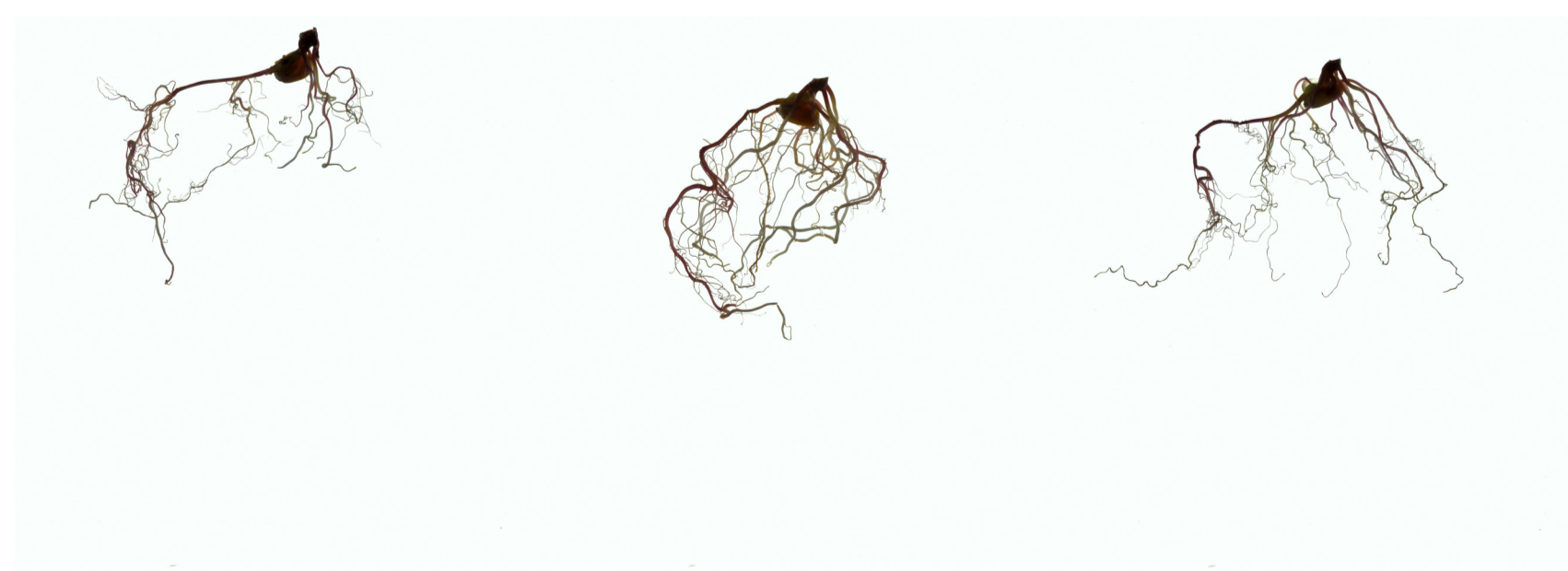CK1+1  $\mu\text{M}$ 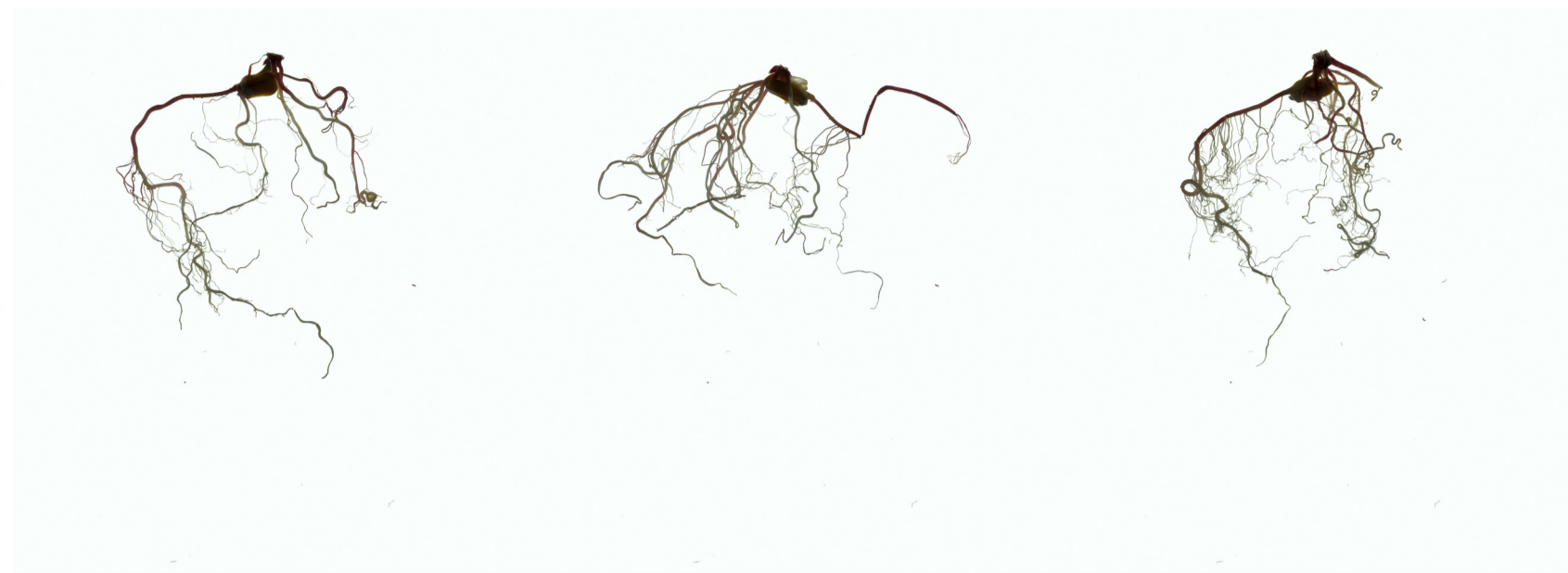CK1+0.1  $\mu\text{M}$ 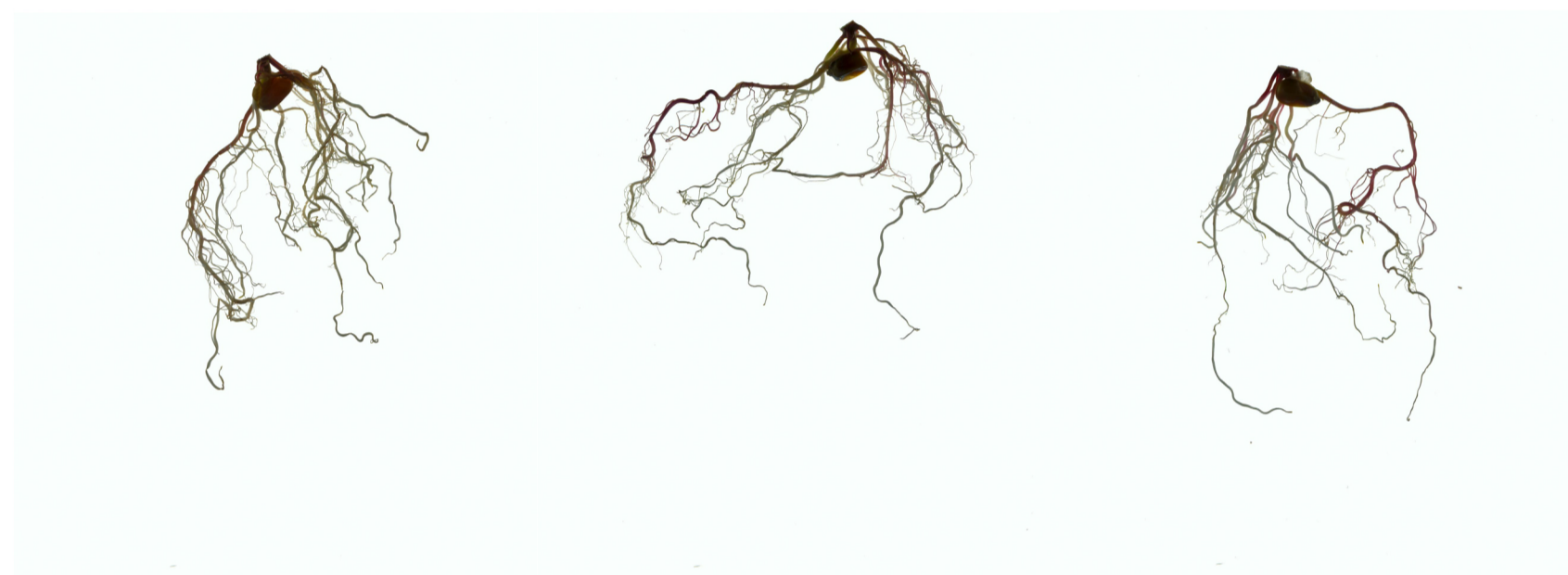CK1+0.01  $\mu\text{M}$ 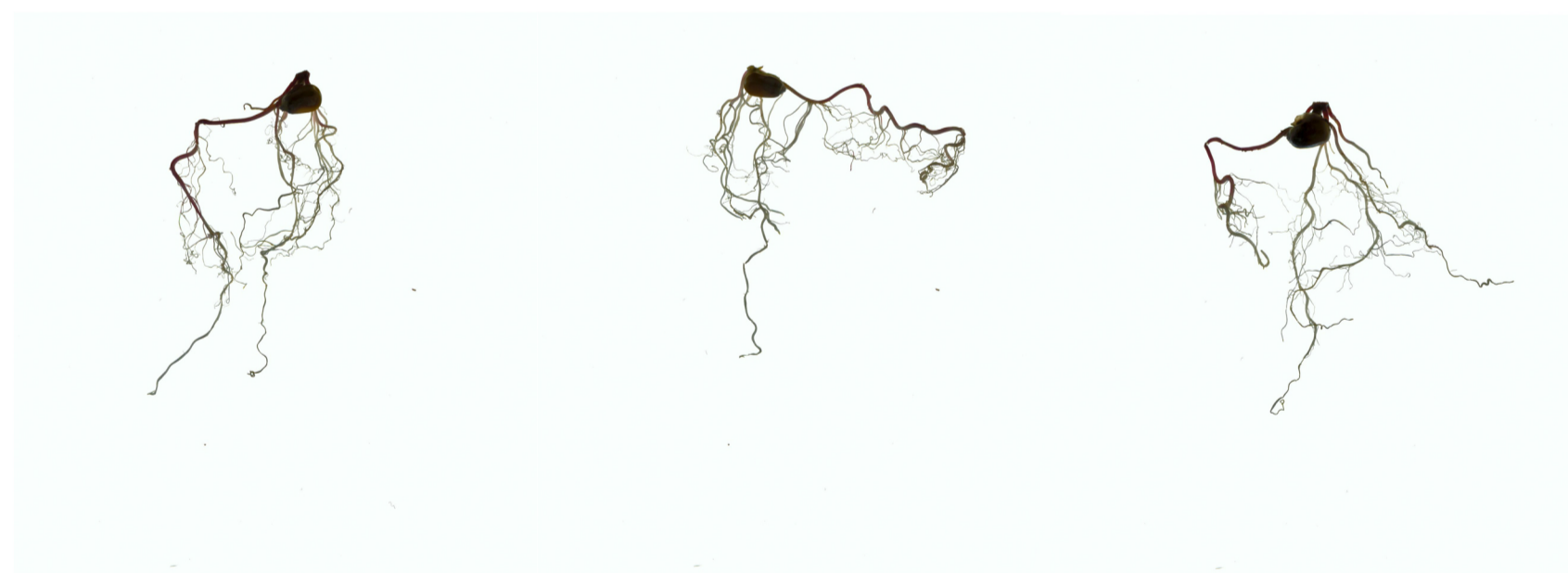CK1+0.001  $\mu\text{M}$ 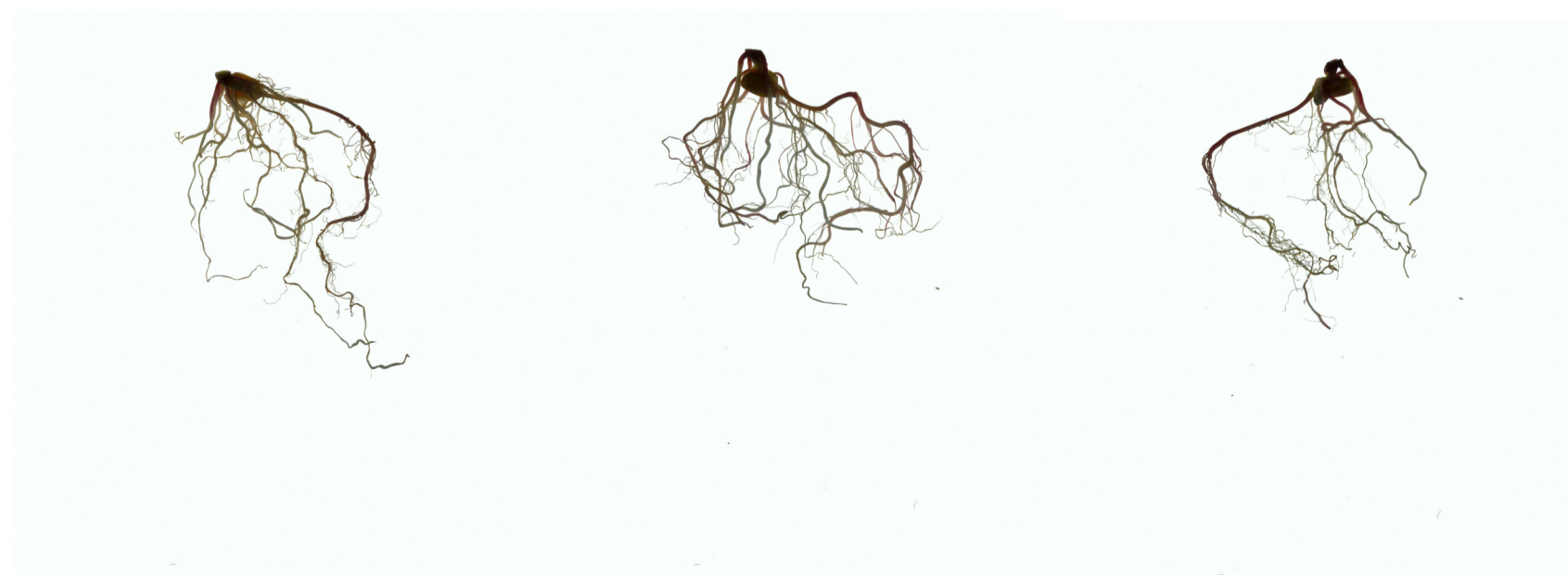

B

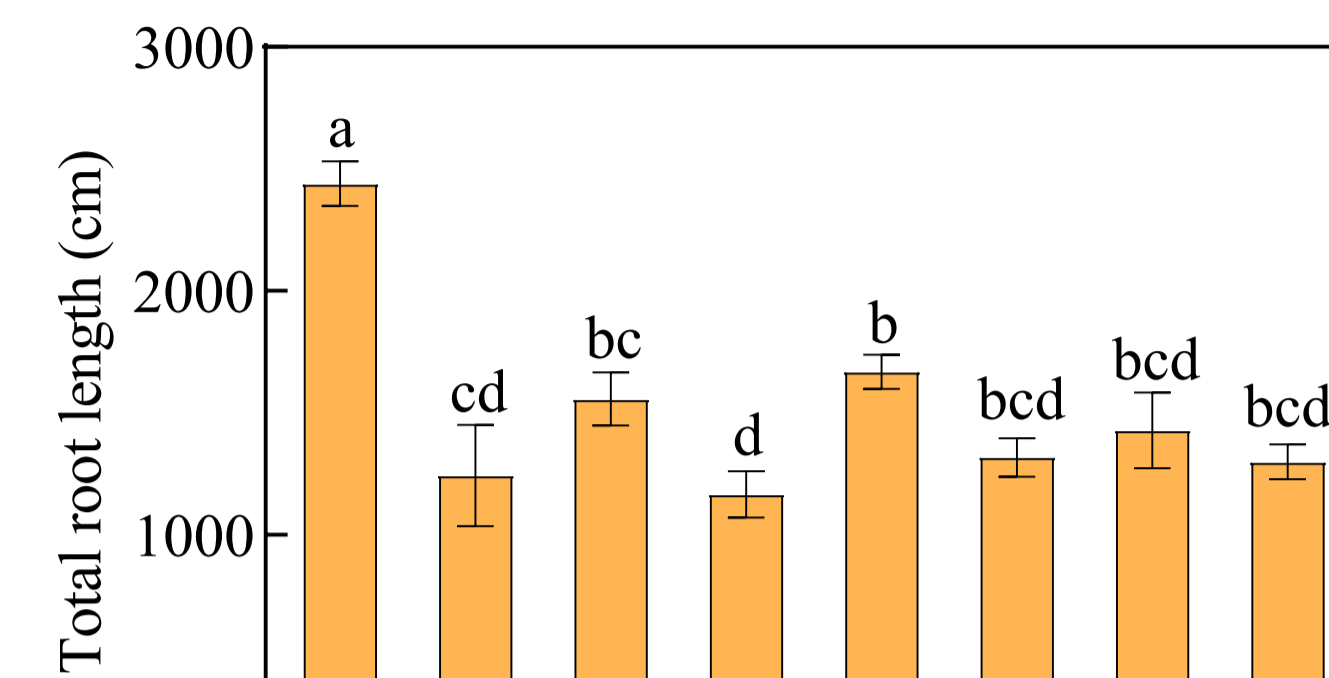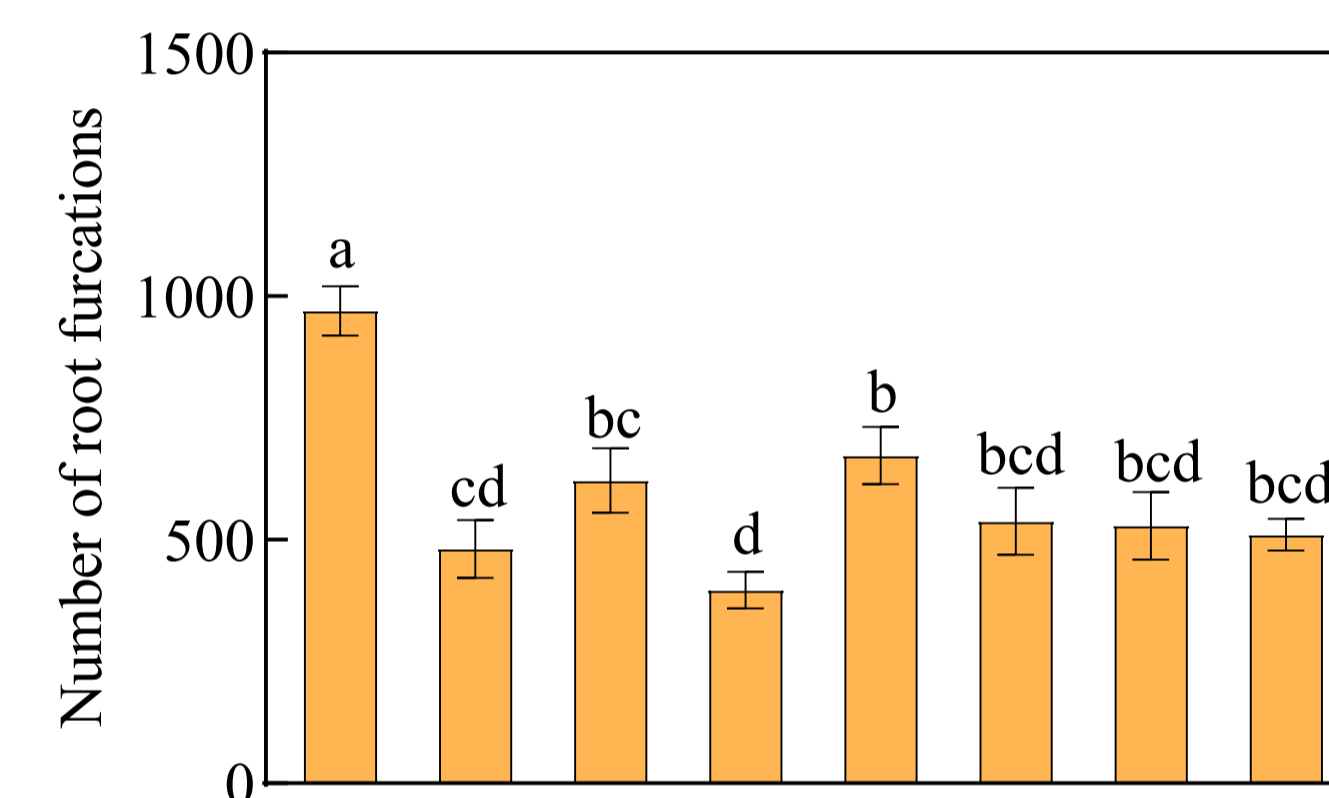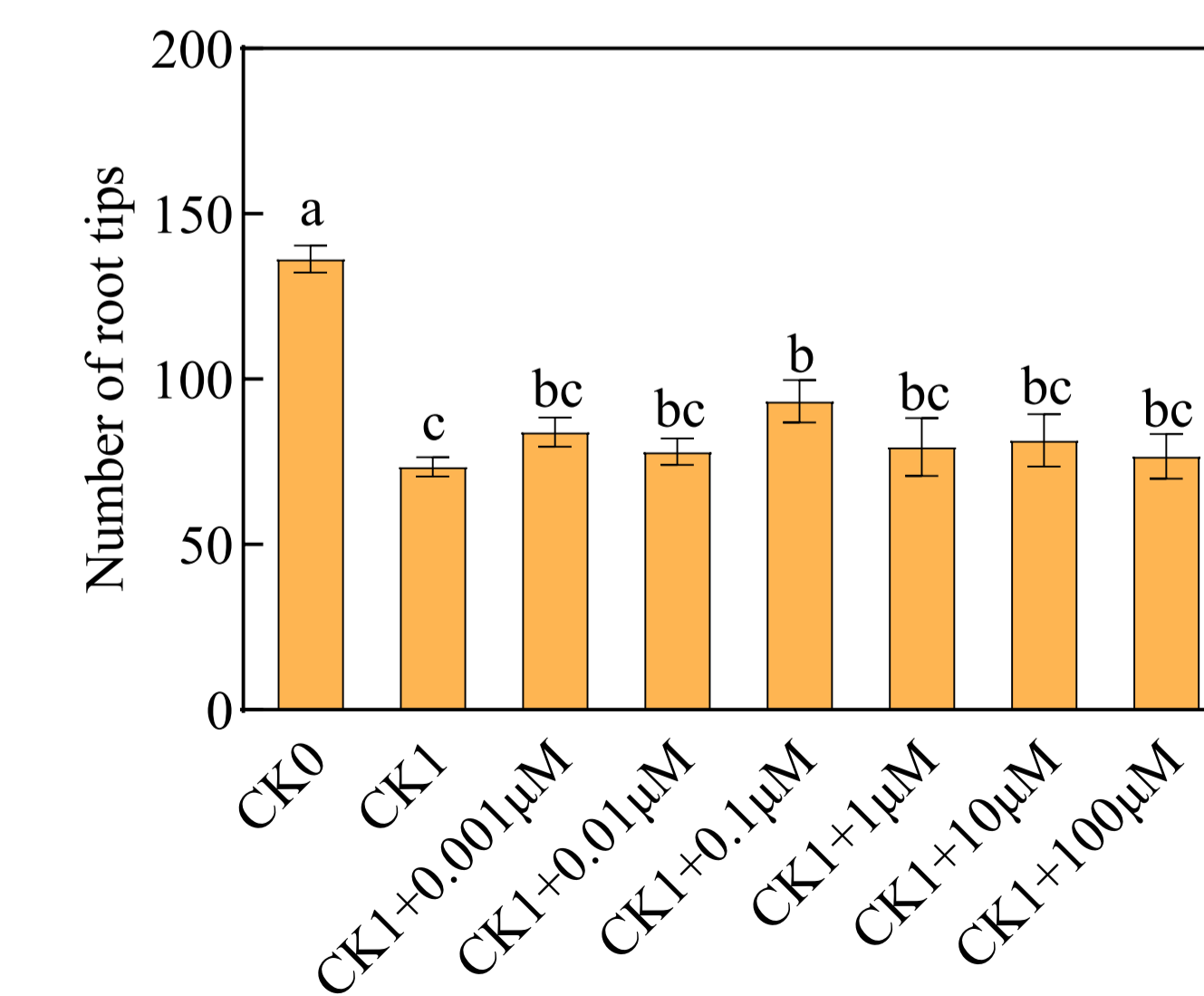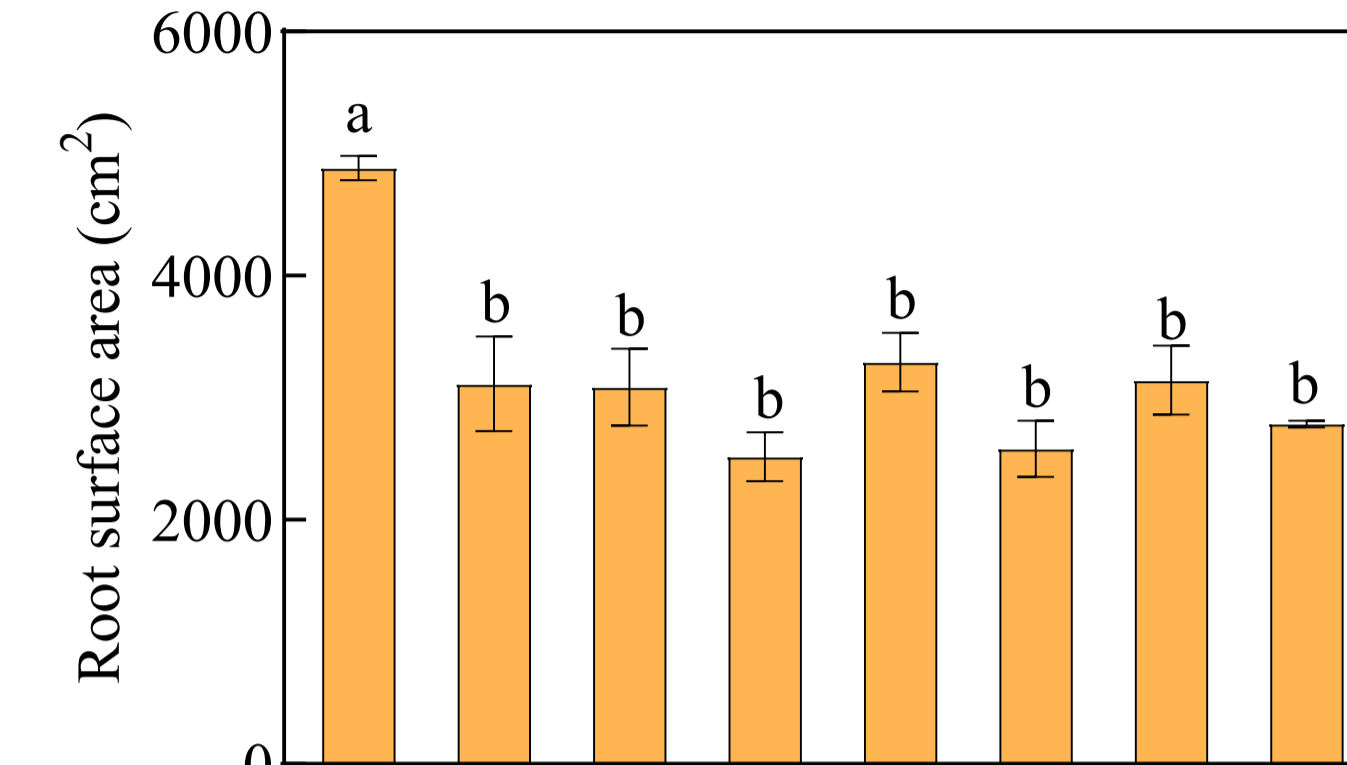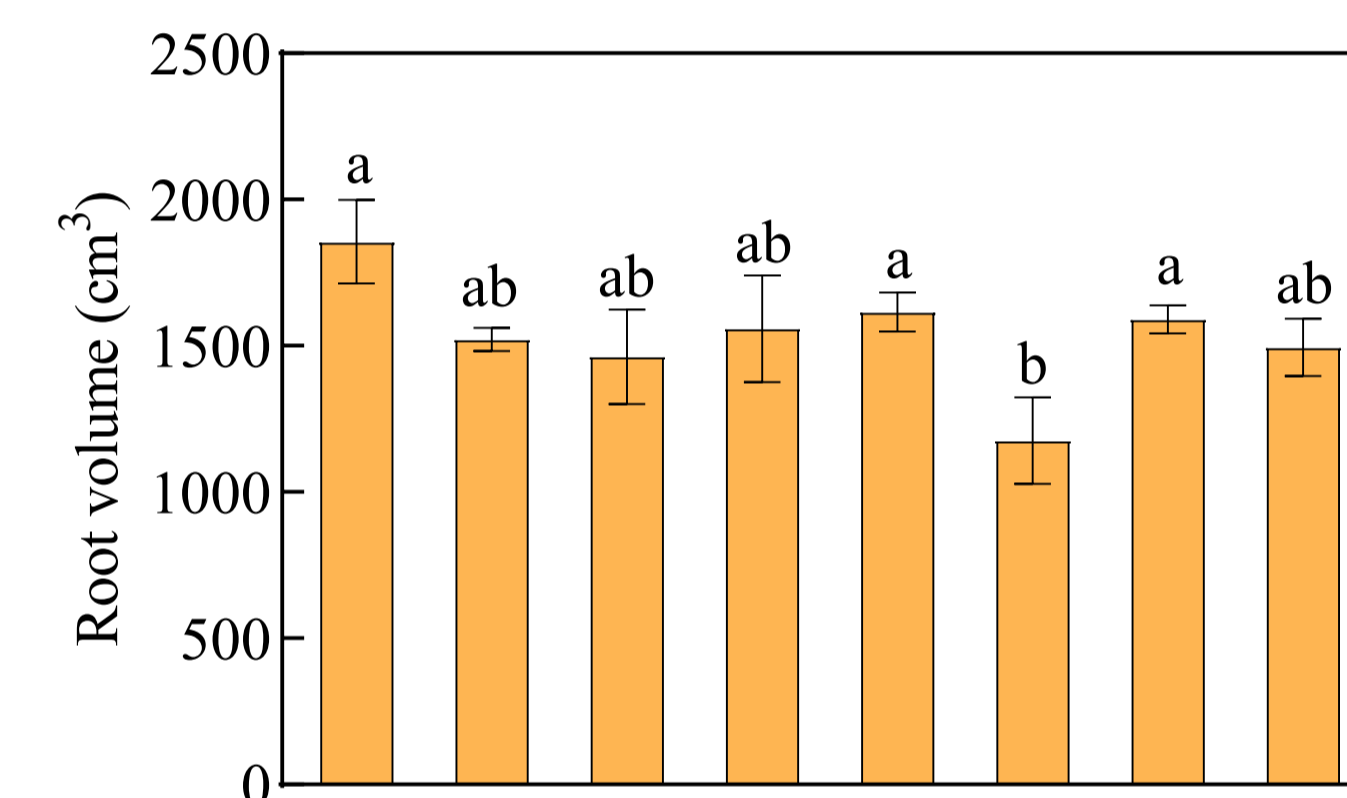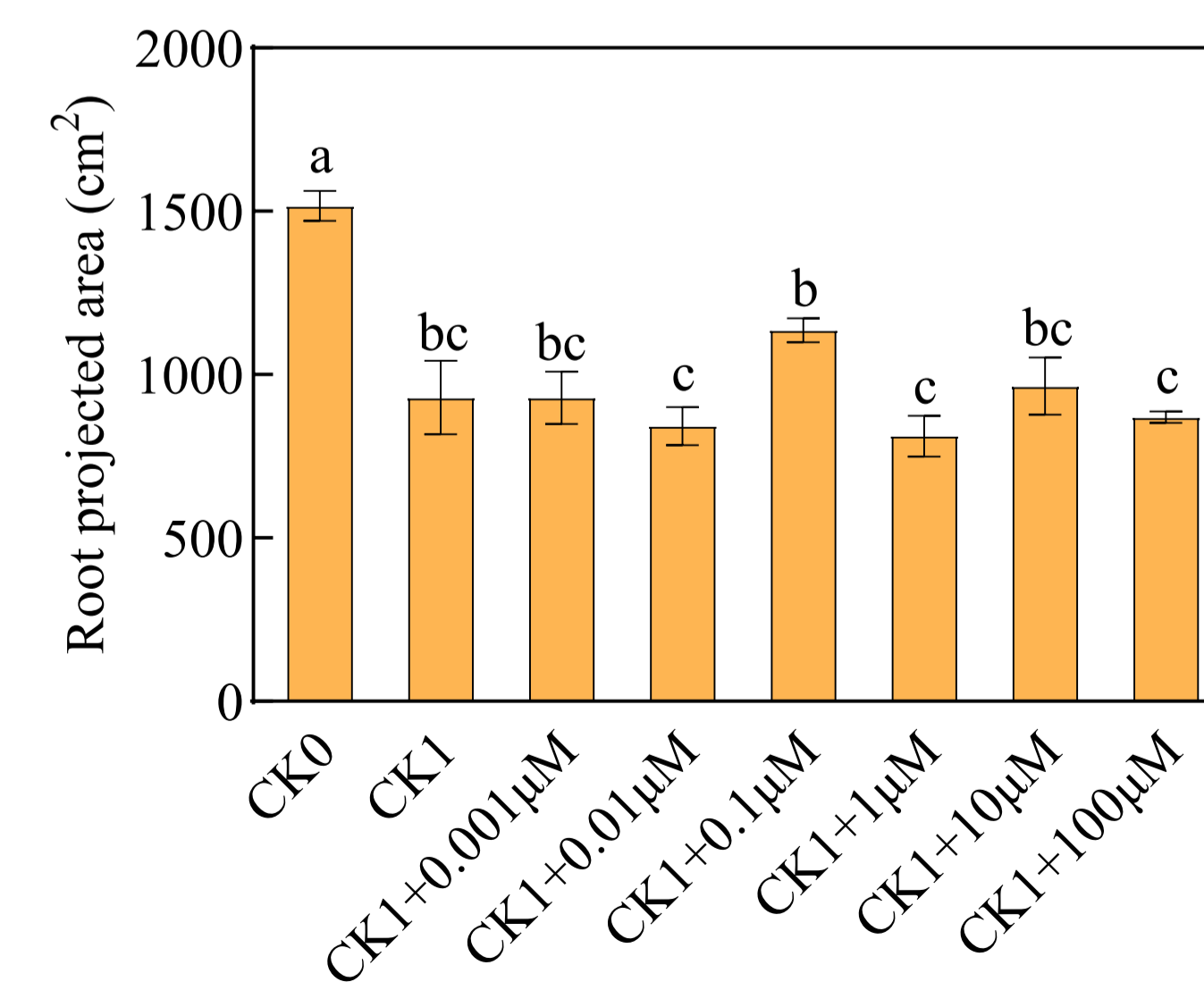

Supplement: Supplementary file 8 [file DataSheet_1.pdf]

A

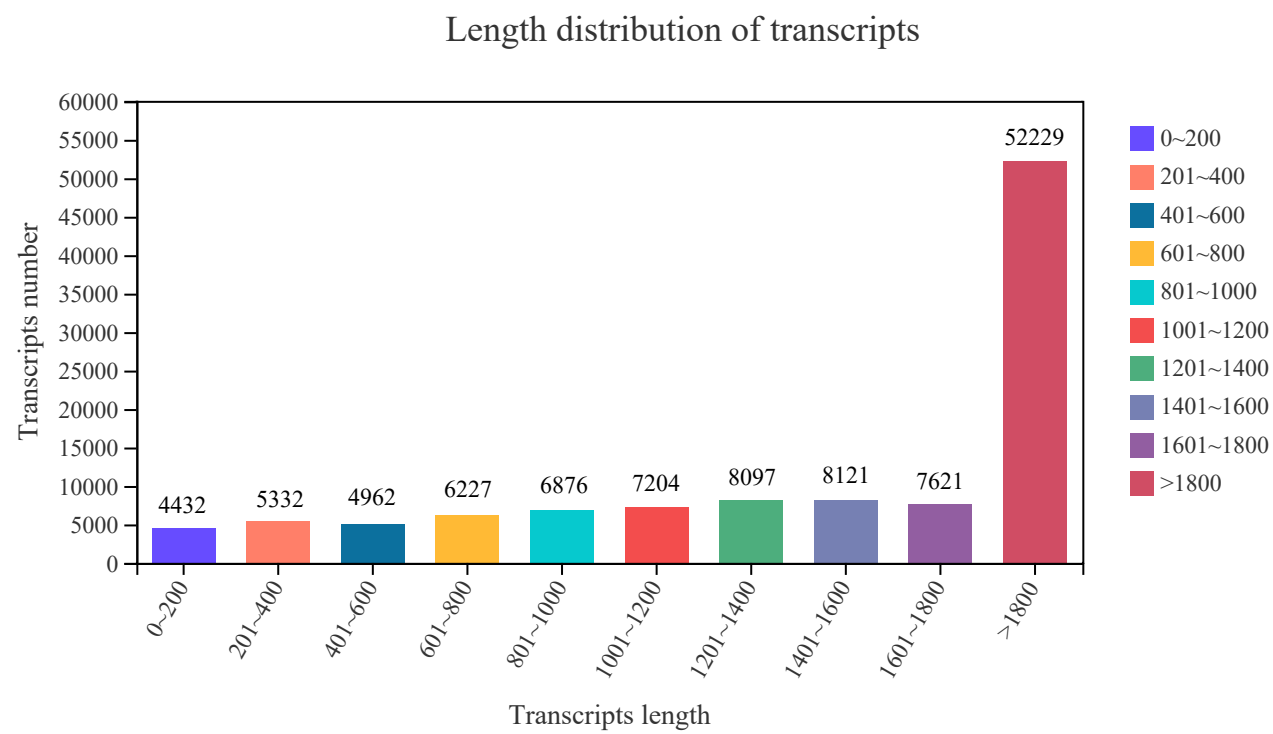

B

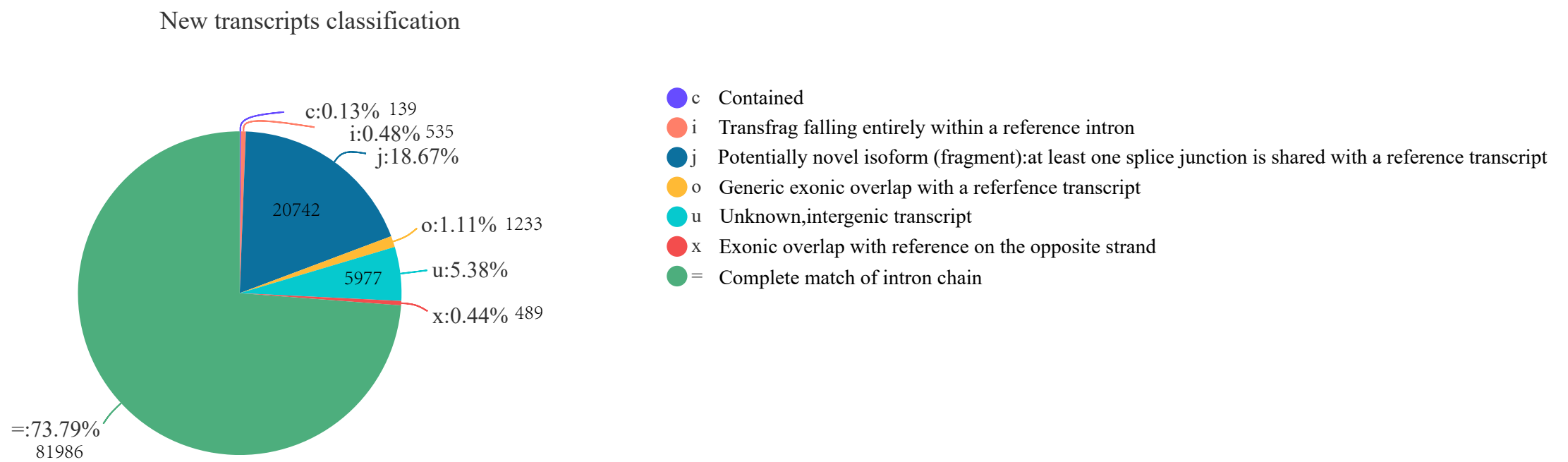

Supplement: Supplementary file 9 [file DataSheet_2.pdf]
